# Supplementary material for: STARRPeaker: uniform processing and accurate identification of STARR-seq active regions
Source: Genome Biol. 2020 Dec 8;21:298. doi: 10.1186/s13059-020-02194-x (PMC7722316; doi:10.1186/s13059-020-02194-x)
Supplement: Supplementary file 1 — Additional file 1: Supplementary Figure S1. Comparison of STARR-seq output coverage calculated using the center of the fragment to using the start position of the sequencing read. (A) Distribution of shift in final peak locations resulting from using two alternative coverage counting schemes in HepG2. Comparison of (B) overall fold enrichment level, (C) P value, and (D) size of resulting peaks. Supplementary Figure S2. (Shadow figure of Fig. 2) Correlation of STARR-seq output coverage with covariates (STARR-seq input coverage, GC content, mappability, and RNA structure) in various STARR-seq datasets, including (A) K562, (B) HeLa-S3 untreated WG-STARR-seq (Muerdter 2018), (C) GM12878 HiDRA ATAC-STARR-seq (Wang 2018), and (D) K562 Cap-STARR-seq (Rathert 2015). PCC: Pearson Correlation Coefficient. Supplementary Figure S3. Contribution of covariates and model selection. (A) Q-Q plots of residuals for various models with different sets of covariates showing the goodness of fit. (B) Both AIC and BIC measure relative qualities of statistical models considering the trade-off between the goodness of fit and the simplicity of the model. DNA: genomic DNA fragment coverage; GC: GC-bias; MAP: mappability, FOLD: folding free energy; AIC: Akaike information criterion; BIC: Bayesian information criterion. Supplementary Figure S4. (Shadow figure of Fig. 3) Fitting of various STARR-seq datasets using three distribution models: negative binomial, binomial, and Poisson. Datasets included (A) K562, (B) HeLa-S3 untreated WG-STARR-seq (Muerdter 2018), (C) GM12878 HiDRA ATAC-STARR-seq (Wang 2018), and (D) K562 Cap-STARR-seq (Rathert 2015). Supplementary Figure S5. Q-Q plots of the P value distribution. (A) HepG2 and (B) K562. The red line is a reference line where the expected P values match the observed ones. Supplementary Figure S6. Correlation of RNA fragment coverage between replicates for (A) HepG2 or (B) K562 cell lines. Pairwise coverage was calculated using 1000 bp bins across the [file 13059_2020_2194_MOESM1_ESM.pdf]

Figure S1

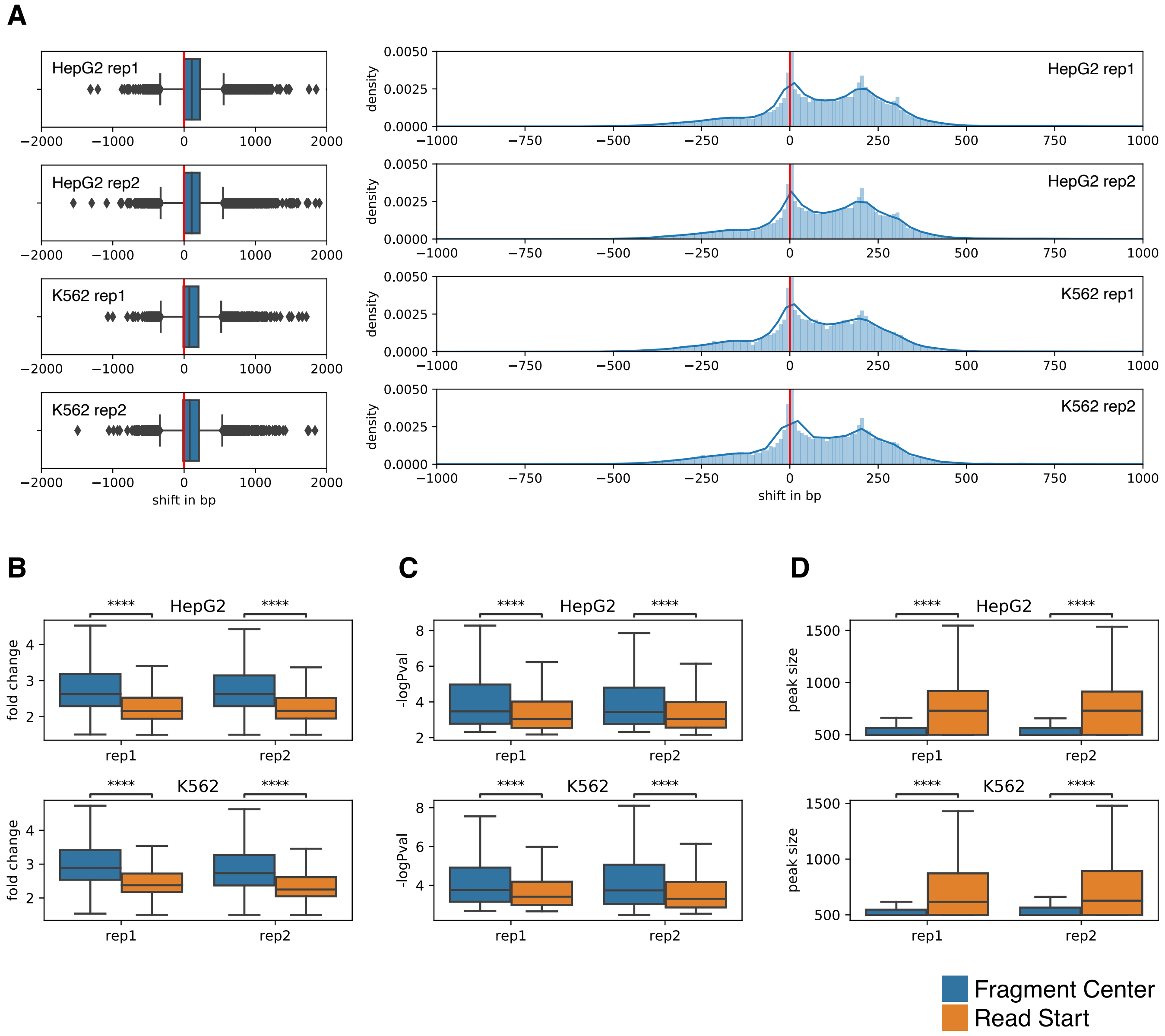
A
HepG2 rep1
HepG2 rep2
K562 rep1
K562 rep2
shift in bp
density
B
C
D
HepG2
K562
fold change
-logPval
peak size
rep1
rep2
****
Fragment Center
Read Start

Figure S2

A K562 ORI-WG-STARR-seq

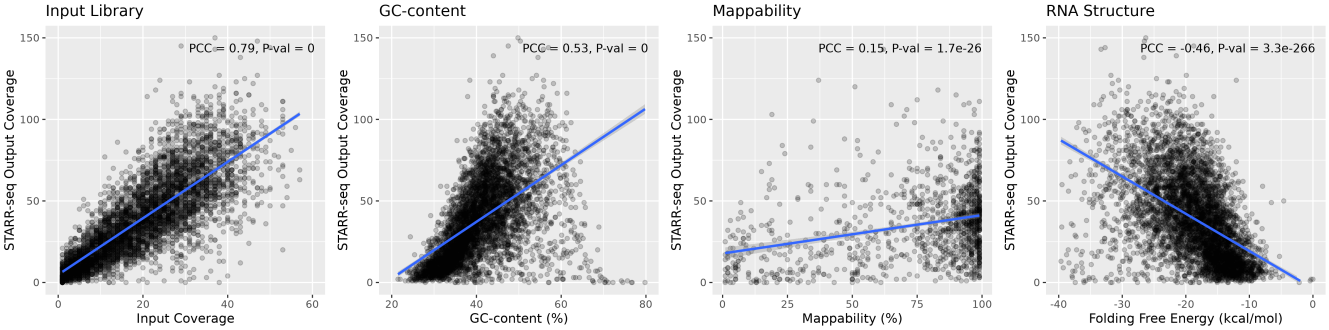

B HeLa-S3 untreated WG-STARR-seq (Muerdter 2018)

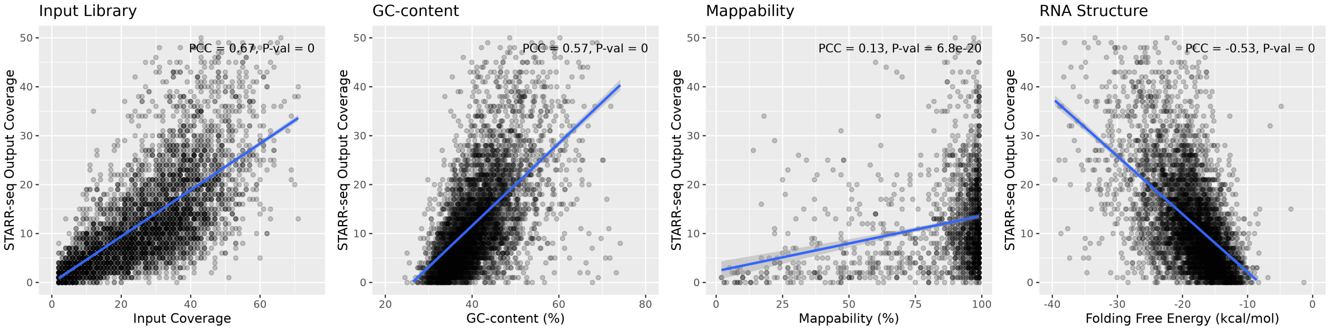

C GM12878 HiDRA ATAC-STARR-seq (Wang 2018)

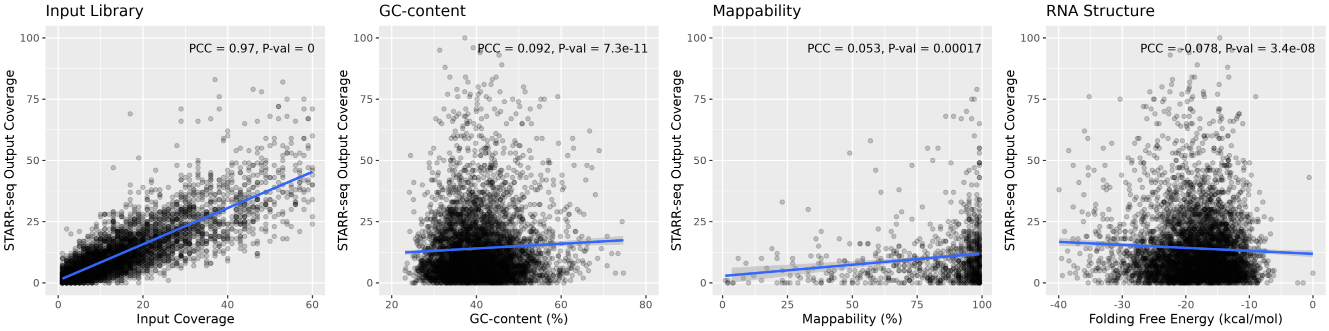

D K562 Cap-STARR-seq (Rathert 2015)

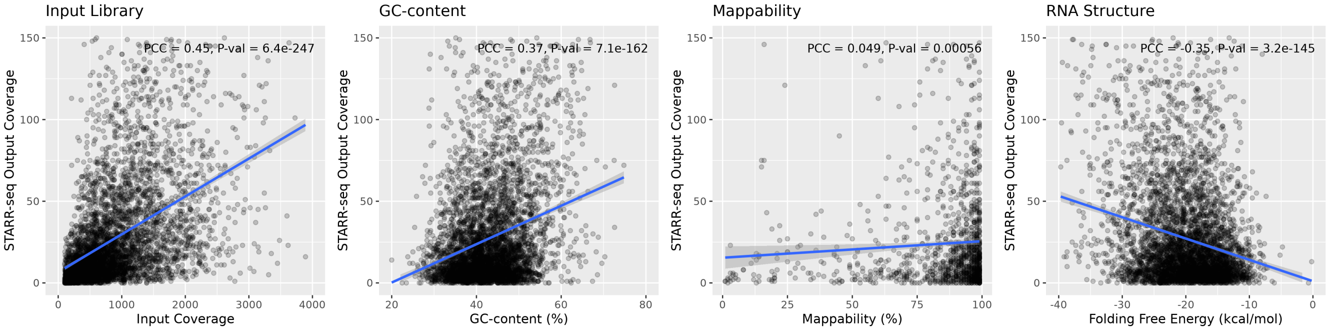

Figure S3

A

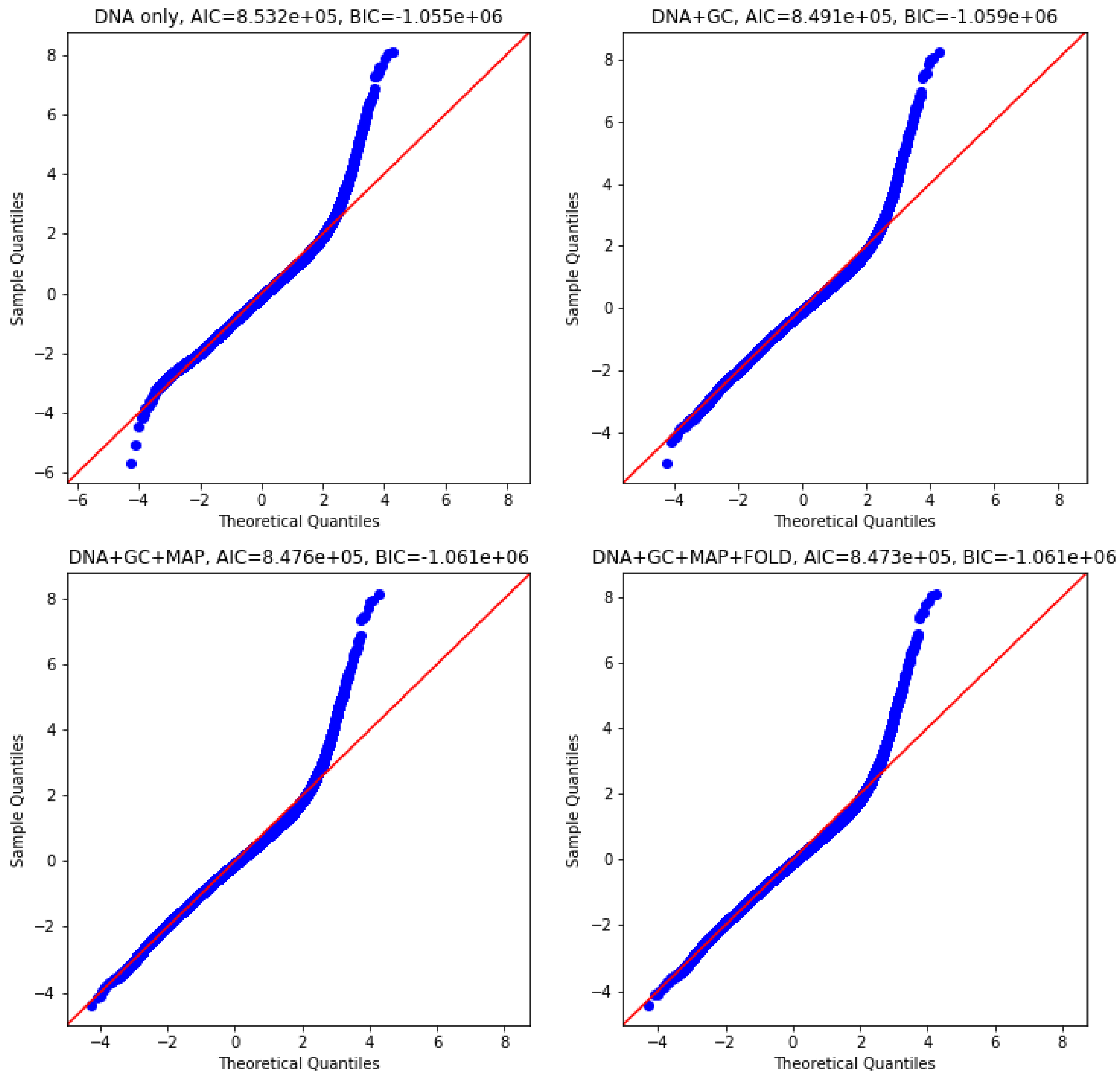
DNA only, AIC=8.532e+05, BIC=-1.055e+06
DNA+GC, AIC=8.491e+05, BIC=-1.059e+06
DNA+GC+MAP, AIC=8.476e+05, BIC=-1.061e+06
DNA+GC+MAP+FOLD, AIC=8.473e+05, BIC=-1.061e+06
Sample Quantiles
Theoretical Quantiles

B

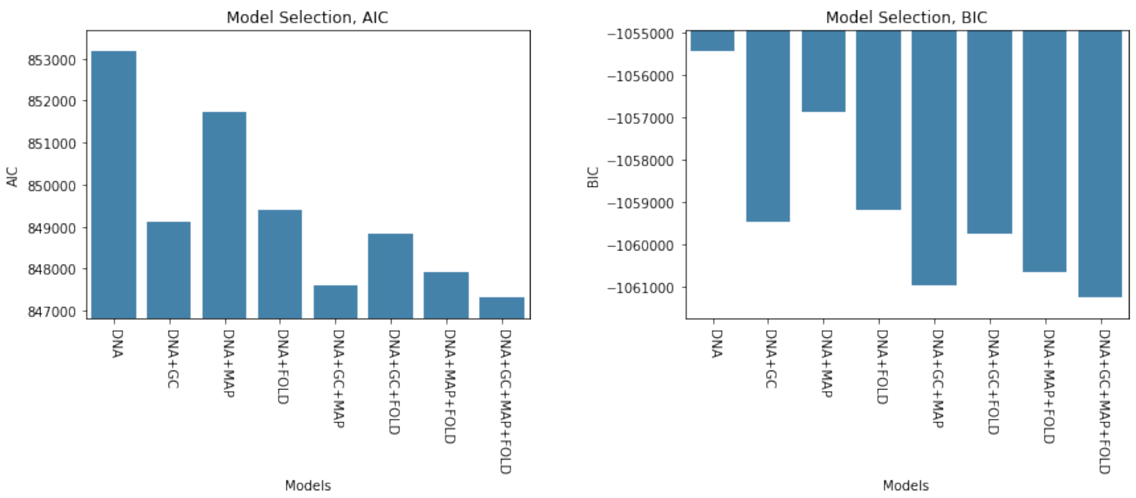
Model Selection, AIC
AIC
853000
852000
851000
850000
849000
848000
847000
Model Selection, BIC
BIC
−1055000
−1056000
−1057000
−1058000
−1059000
−1060000
−1061000
DNA
DNA+GC
DNA+MAP
DNA+FOLD
DNA+GC+MAP
DNA+GC+FOLD
DNA+MAP+FOLD
DNA+GC+MAP+FOLD
Models

Figure S4

A K562 ORI-WG-STARR-seq

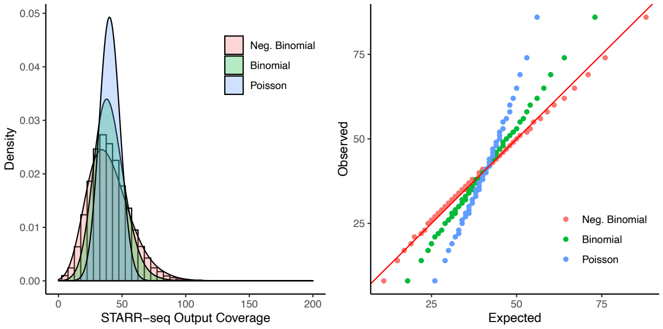

B HeLa-S3 untreated WG-STARR-seq (Muerdter 2018)

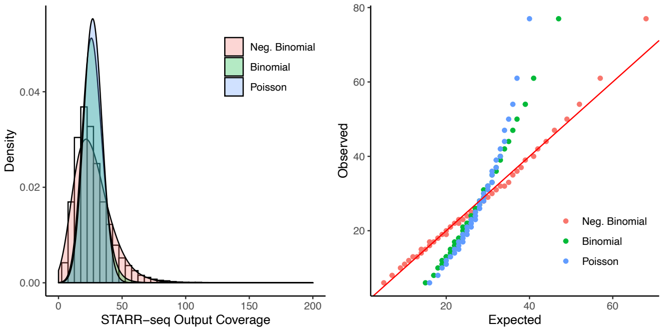

C GM12878 HiDRA ATAC-STARR-seq (Wang 2018)

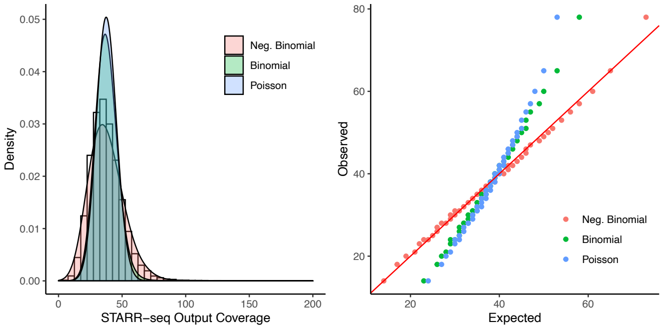

D K562 Cap-STARR-seq (Rathert 2015)

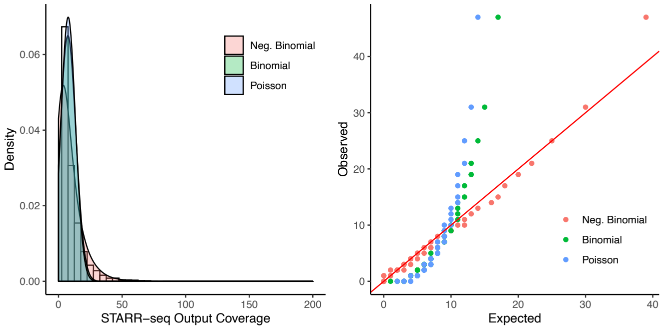

Figure S5

A

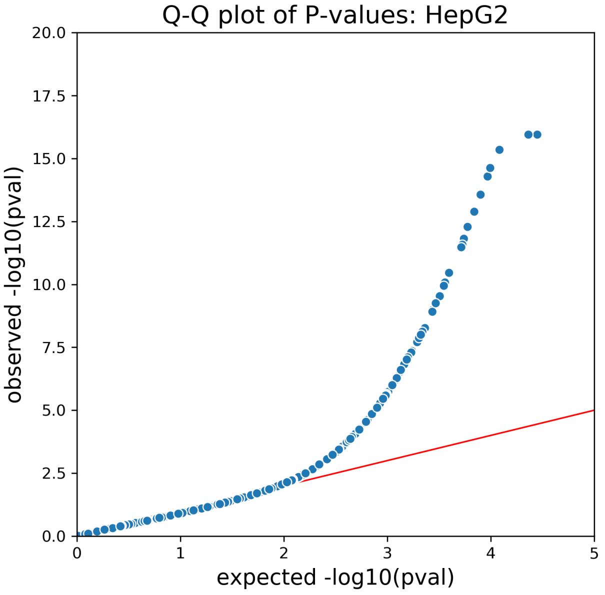
Q-Q plot of P-values: HepG2
observed -log10(pval)
expected -log10(pval)
0.0
2.5
5.0
7.5
10.0
12.5
15.0
17.5
20.0
0
1
2
3
4
5

B

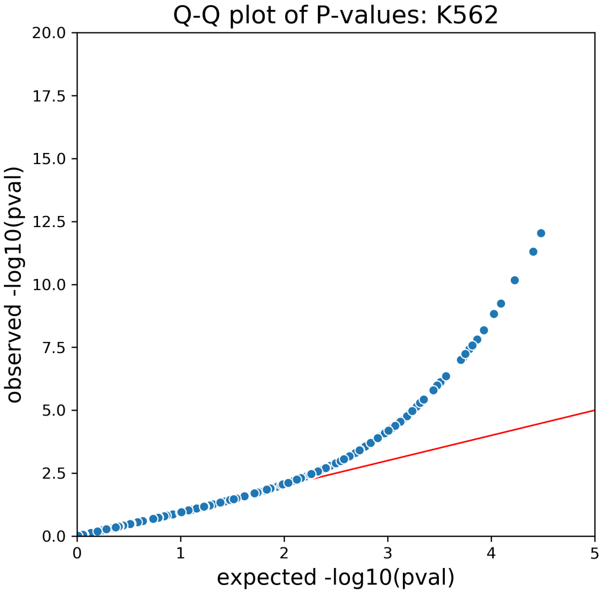
Q-Q plot of P-values: K562
observed -log10(pval)
expected -log10(pval)
0.0
2.5
5.0
7.5
10.0
12.5
15.0
17.5
20.0
0
1
2
3
4
5

Figure S6

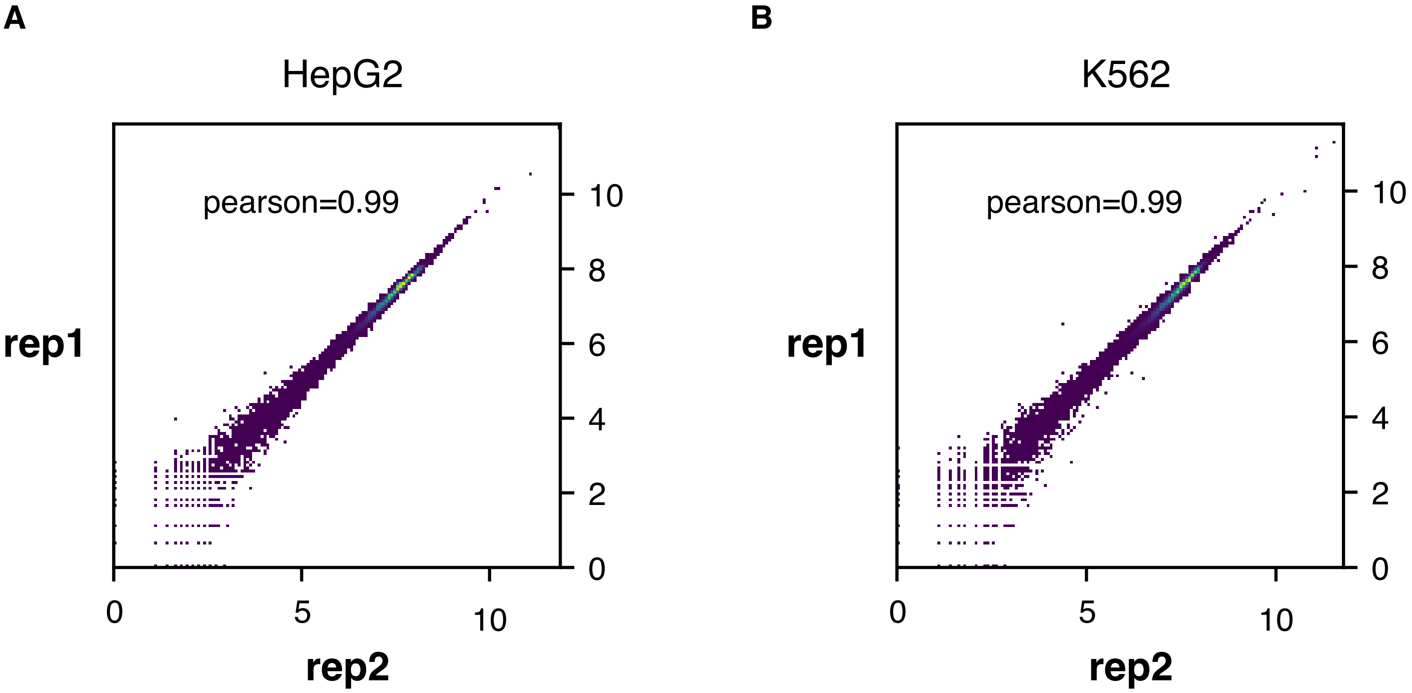
A
B
HepG2
K562
pearson=0.99
pearson=0.99
rep1
rep1
rep2
rep2
0
5
10
0
2
4
6
8
10

Figure S7

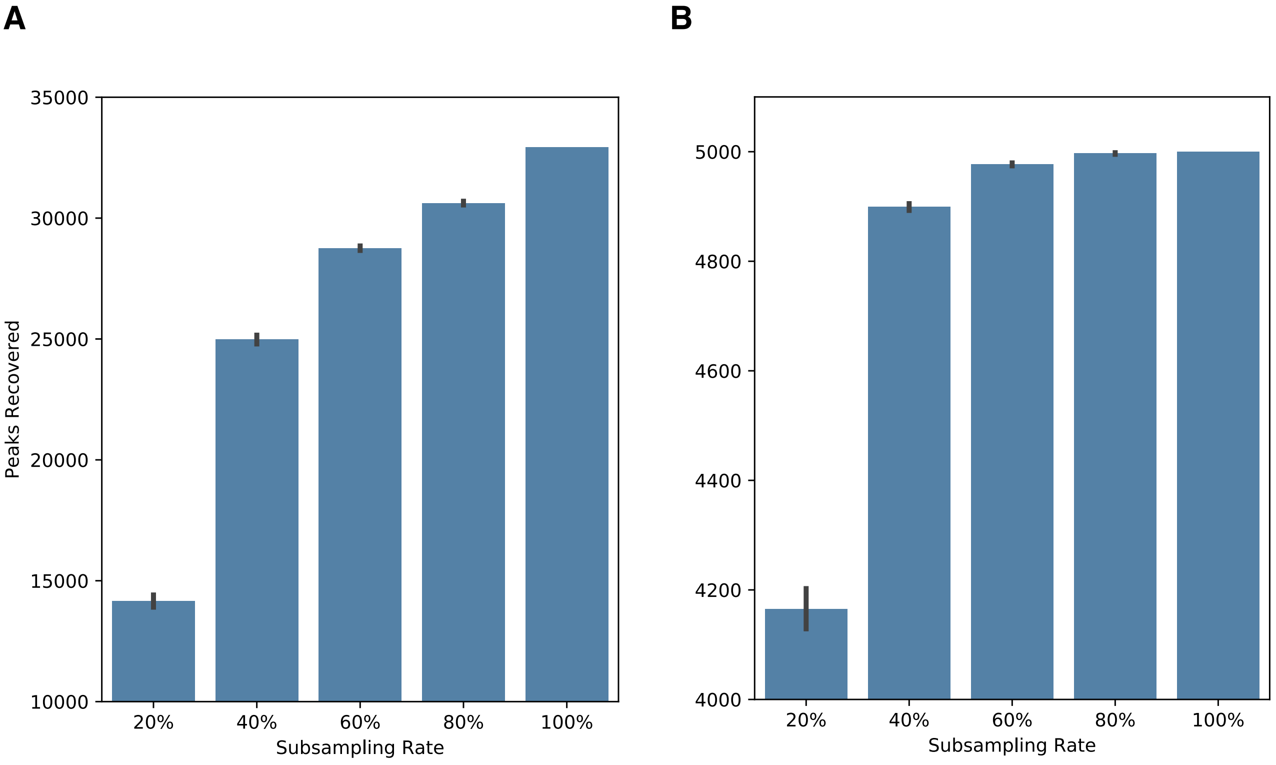
A
B
35000
30000
25000
20000
15000
10000
Peaks Recovered
20%
40%
60%
80%
100%
Subsampling Rate
5000
4800
4600
4400
4200
4000
20%
40%
60%
80%
100%
Subsampling Rate

Figure S8

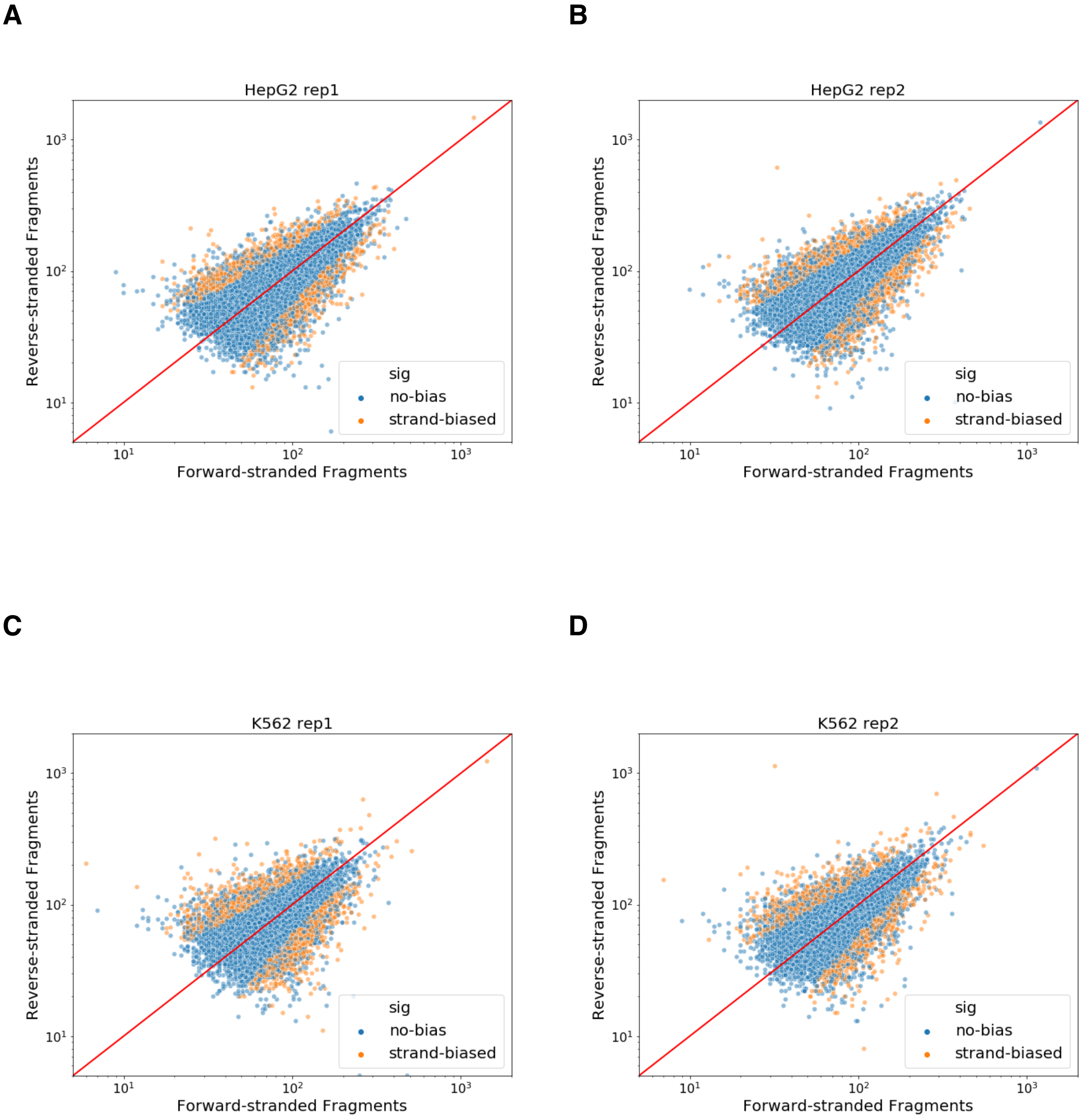
A
B
HepG2 rep1
HepG2 rep2
Reverse-stranded Fragments
Forward-stranded Fragments
sig
no-bias
strand-biased
C
D
K562 rep1
K562 rep2
Reverse-stranded Fragments
Forward-stranded Fragments
sig
no-bias
strand-biased

Figure S9

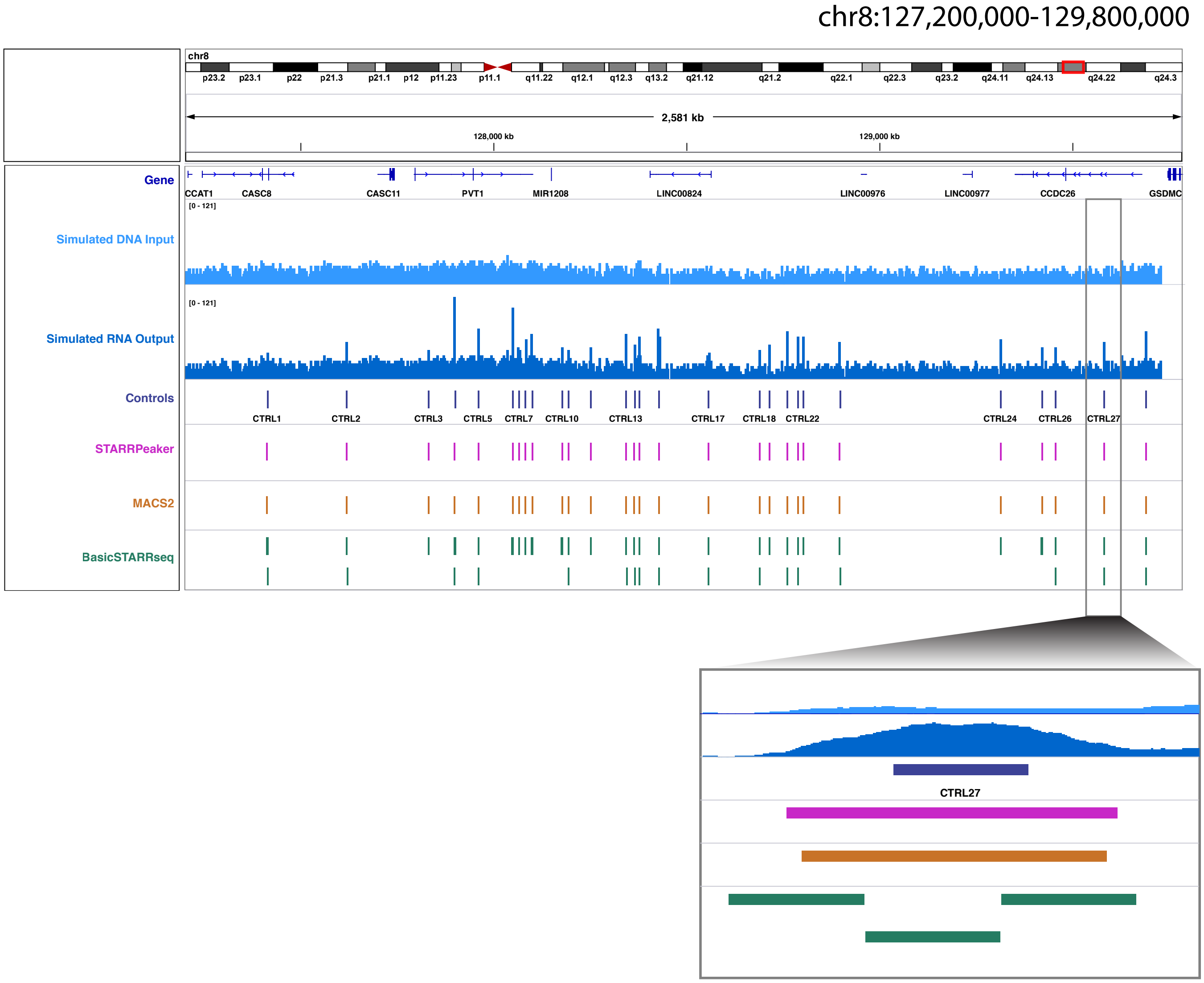
chr8:127,200,000-129,800,000
chr8
p23.2 p23.1 p22 p21.3 p21.1 p12 p11.23 p11.1 q11.22 q12.1 q12.3 q13.2 q21.12 q21.2 q22.1 q22.3 q23.2 q24.11 q24.13 q24.22 q24.3
2,581 kb
128,000 kb
129,000 kb
Gene
CCAT1 CASC8 CASC11 PVT1 MIR1208 LINC00824 LINC00976 LINC00977 CCDC26 GSDMC
Simulated DNA Input
[0 - 121]
Simulated RNA Output
[0 - 121]
Controls
CTRL1 CTRL2 CTRL3 CTRL5 CTRL7 CTRL10 CTRL13 CTRL17 CTRL18 CTRL22 CTRL24 CTRL26 CTRL27
STARRPeaker
MACS2
BasicSTARRseq
CTRL27

Figure S10

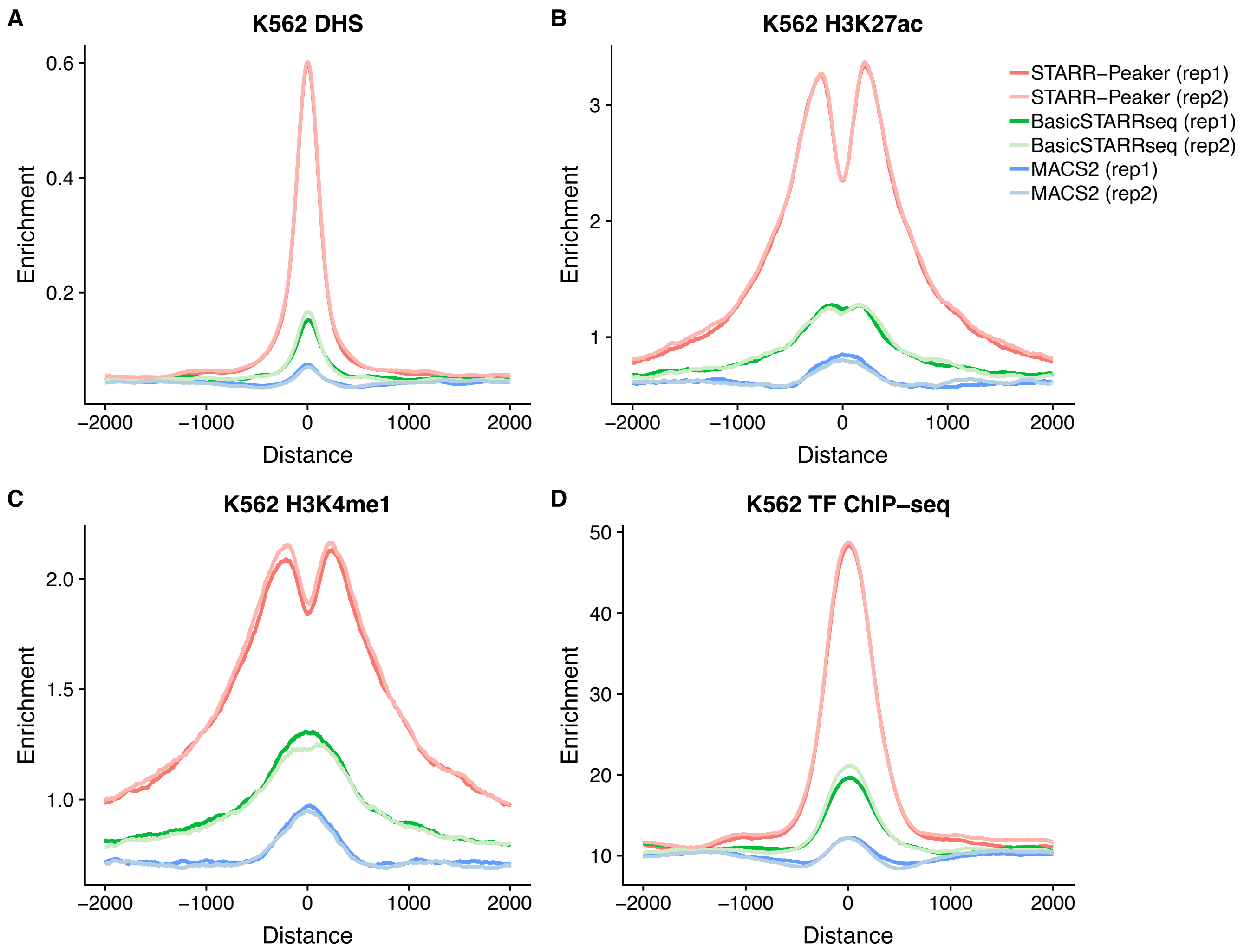

A
K562 DHS
B
K562 H3K27ac
STARR−Peaker (rep1)
STARR−Peaker (rep2)
BasicSTARRseq (rep1)
BasicSTARRseq (rep2)
MACS2 (rep1)
MACS2 (rep2)
C
K562 H3K4me1
D
K562 TF ChIP−seq
Enrichment
Distance

Figure S11

A GATA1 locus chrX:48774179-48806695

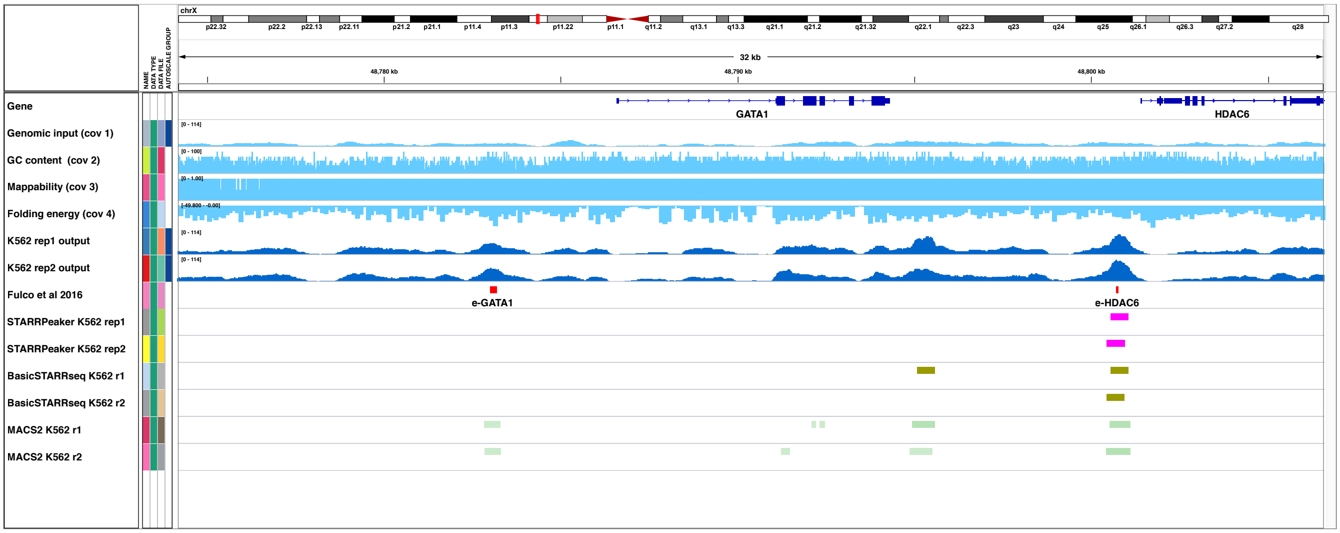

B MYC locus chr8:127830000-128090000

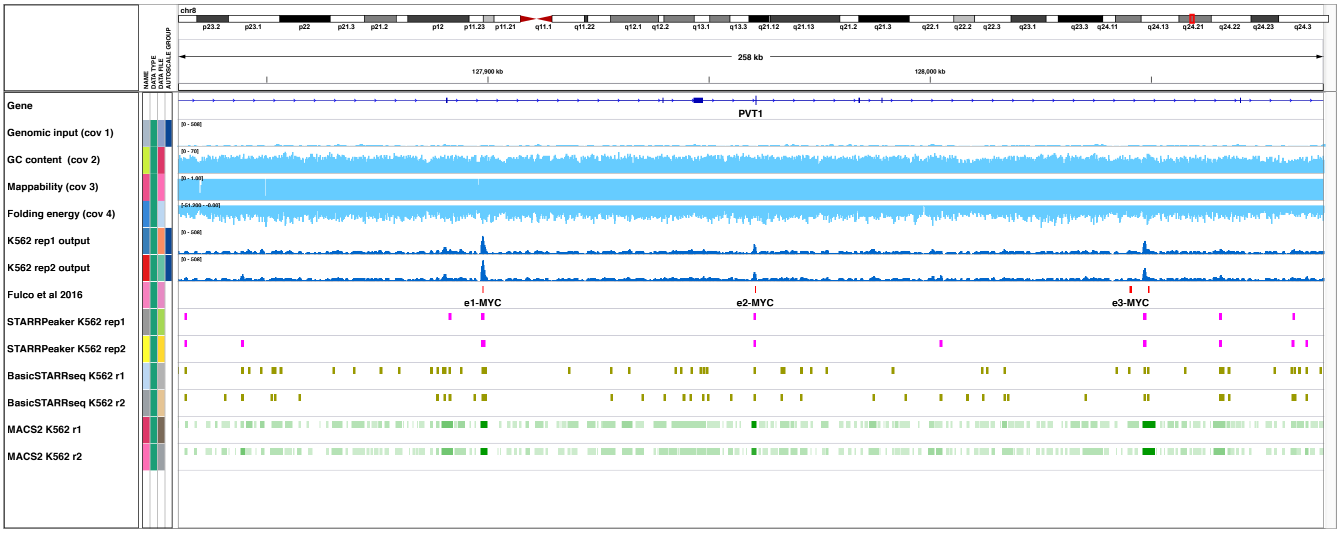

C MYC locus chr8:129550000-129720000

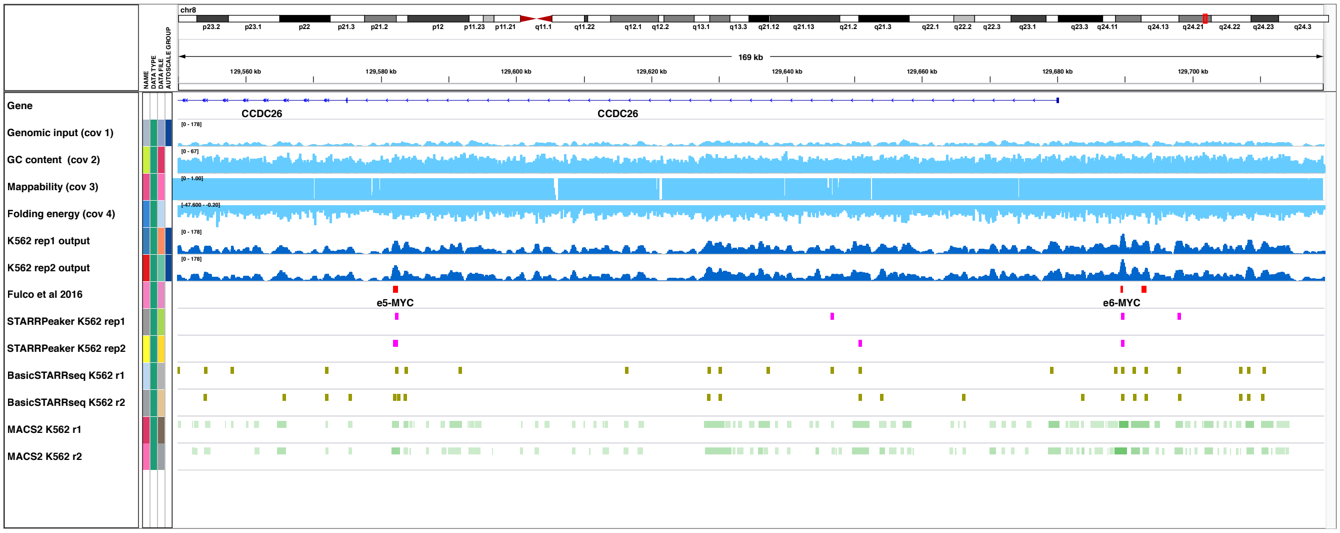

Figure S12

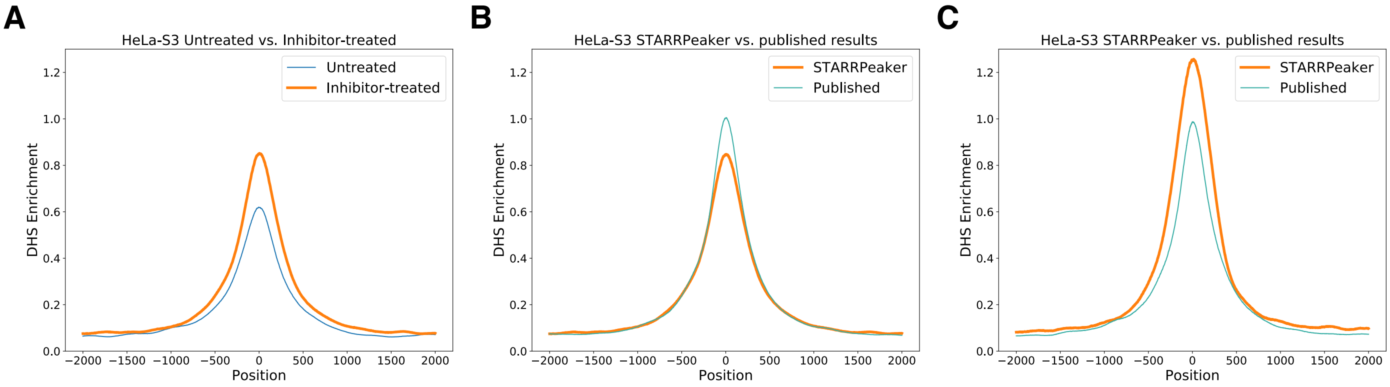
A
HeLa-S3 Untreated vs. Inhibitor-treated
Untreated
Inhibitor-treated
DHS Enrichment
Position
B
HeLa-S3 STARRPeaker vs. published results
STARRPeaker
Published
DHS Enrichment
Position
C
HeLa-S3 STARRPeaker vs. published results
STARRPeaker
Published
DHS Enrichment
Position

Figure S13

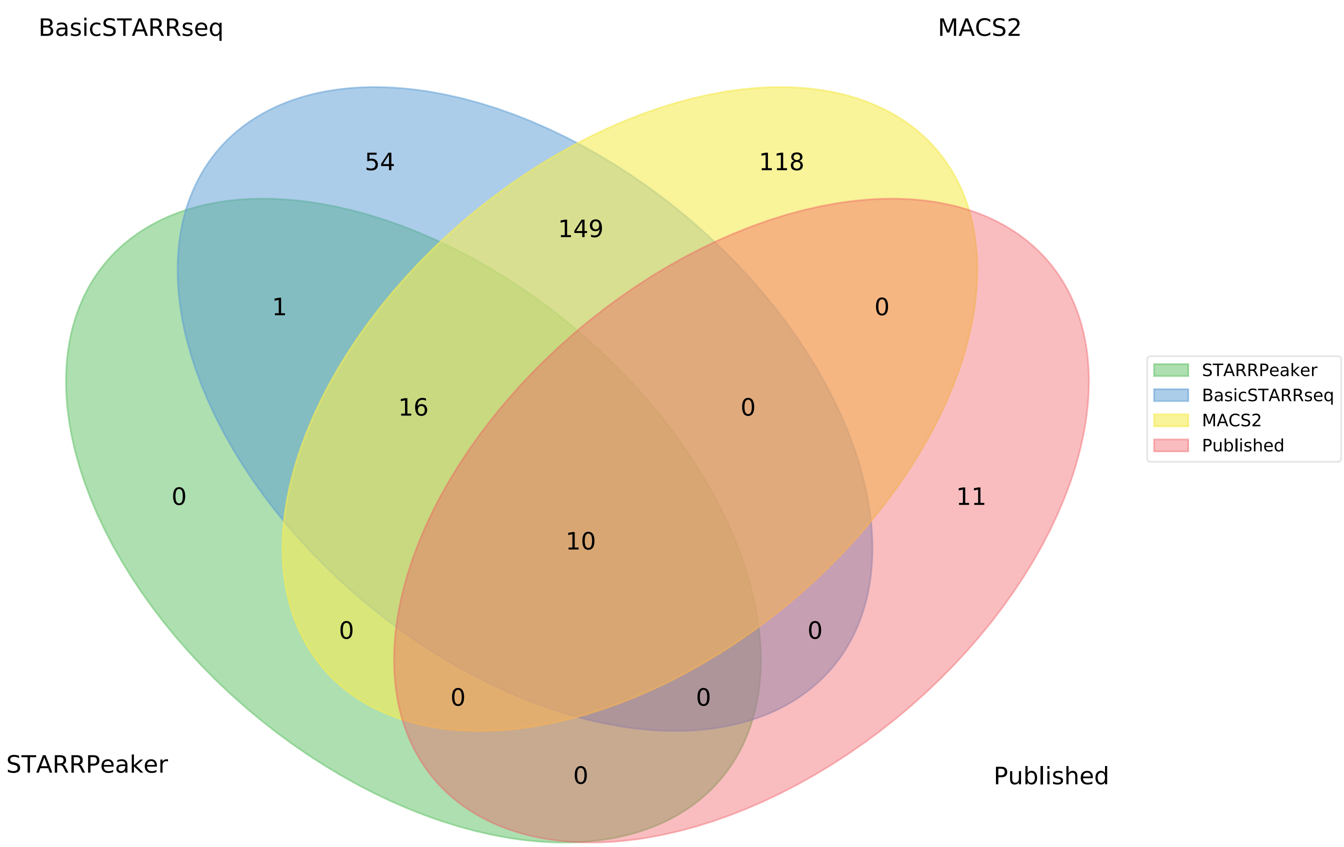

BasicSTARRseq
MACS2
54
118
149
1
0
16
0
0
11
10
0
0
0
0
STARRPeaker
Published
0
STARRPeaker
BasicSTARRseq
MACS2
Published
