# Supplementary material for: STARRPeaker: uniform processing and accurate identification of STARR-seq active regions
Source: Genome Biol. 2020 Dec 8;21:298. doi: 10.1186/s13059-020-02194-x (PMC7722316; doi:10.1186/s13059-020-02194-x)
Supplement: Supplementary file 10 — Additional file 10. Review history. [file 13059_2020_2194_MOESM10_ESM.docx]

**Review history**

**First round of review**

**Reviewer 1**

In this study, Lee and col. developed a bioinformatic pipeline (STARRPeaker ) to identify enhancers regions based on high-throughput reporter assays, namely STARR-seq approaches. The processing and analyses of STARR-seq data are generally difficult tasks because the results can be confounded by several potential biases which are not fully addressed by current pipelines. Given the recent interest in high-throughput reporter assays, the development of an accurate pipeline to identify bona fide enhancers is of major interest in the field of functional genomics. Moreover this study also provided whole genome STARR-seq experiments in two human cell lines which are used to validate the STARRPeaker method, but can also represent a valuable resource if properly validated.  


Specific comments
1.    The STARRPeaker model introduced several covariates to adjust the STARR-seq signal to account for potential biases, including fragment coverage of input genomic libraries, GC content, mappability, and the thermodynamic stability of genomic libraries. Another potential bias might come from the relative orientation of the fragments. Thus, the authors should explore the orientation consistency of their datasets and eventually adjust their method to account for potential orientation-dependent biases.

2.    STARR-seq experiments were performed in duplicate. The overall correlation between the replicates should be shown. Besides, the overlap between the enhancers identified in each replicate should be mentioned.

3.    It will be interesting to assess whether similar findings can be observed using other published whole-genome STARR-seq in mammals (e.g., doi:10.1038/nmeth.4534; DOI 10.1186/s13059-017-1345-5).

4.    To better assess the quality of the resources, the authors should provide several examples of loci comparing the STARR-seq signals and peaks with epigenomic profiles. Whether known enhancers (i.e. independently validated from the literature) are also identified by STARRPeaker should be addressed. For instance, are the enhancers recently identified by CRISPRi in k562 (doi:10.1126/science.aag2445.) identified by the STARRPeaker?

5.    Previous whole-genome STARR-seq experiments in mammals have suggested that a substantial number of active enhancers are found in closed chromatin (doi:10.1038/nmeth.4534; DOI 10.1186/s13059-017-1345-5). Whether this observation is related to false-positive rate of the used pipelines is not clear. Therefore, what is the percentage of active enhancers found in closed chromatin when using the STARRPeaker method?. Whether the results are consistent or not with the previous finding should be discussed.

6.    Many STARR-seq based approaches have been performed with pre-enriched target sequences (e.g. doi: 10.1038/s41467-018-07746-1, doi: 10.1016/j.stem.2018.06.014, DOI: 10.1038/ncomms7905). How the STARRPeaker performs with pre-enriched based reporter assays is an important issue, as these targeted approaches might become very popular.

7.    A table listing the enhancers identified from each experiment should be provided.

8.   Typo: "We also filtered out read  pairs that had a fragment insert size less than 𝑙𝑚𝑎𝑥 and greater than 𝑙𝑚𝑖𝑛.": It should be the opposite

**Reviewer 2**

Lee et al. introduce STARR-Peaker, a peak-calling algorithm designed specifically to analyze STARR-seq data, an increasingly popular method for detecting active enhancer sequences. The authors demonstrate their approach using two STARR-seq datasets generated in this study, and compare STARR-Peaker with several existing methods.
While a method designed for STARR-seq may be of interest to the Genome Biology readership, we feel that the authors do not present sufficiently convincing evidence of the superiority and applicability of their approach. Several aspects of the method and analyses are not clearly explained and the model is not sufficiently evaluated both in terms of the datasets used and the performance analyses


Major comments:

1. Data: The authors perform all evaluations of STARR-seq properties and of their method using two datasets generated by the authors using the same protocol. To demonstrate that the biases are general and the method is generally applicable and superior, additional external datasets should be used.

2. Figure 1:
a. The figure aims to demonstrate the effect of using the fragment center as opposed to
the beginning to associate reads to bins. However, the authors do not establish that this decision is beneficial, only that it potentially changes the peak centers. Even if the change is significant (see below) - it should be demonstrated that it results in more accurate peaks positioning. This can be done, for instance, by running a variant of the model with alternative fragment counting schemes.
b. The figure shows the effect on four regions, but neither the figure nor the text quantify this effect across all regions or make any statements about how consistent or significant it is.
c. The figure uses a bin size of 50bp, but throughout the paper the authors use 500bp. Please clarify.
d. When examining the regions selected, it appears that the shift in the peak center falls within the same 500bp window in all cases, which seems to mean that this shift won't affect any of the downstream analyses. Please comment on how this decision affects the results of the model.

3. Model and Figure 2:
a. The model description should be clarified and simplified. It is not immediately clear from
the text that STARR-Peaker fits a single null model across all genomic bins, and then tests each genomic bin using the null model to compute p-values. Related to that - there seems to be a typo in the p-value equation (line 287).
b. From Figure 2 it looks like the DNA and RNA counts are highly correlated. It may, therefore, be the case that some of the other biases (e.g., GC content, and maybe confusingly enough - RNA structure properties) also confound the DNA data. This means that in the model, it -may- be sufficient to regress only the DNA term. The authors should explore this point and also report the confounding of the DNA.
c. More generally, the authors should better establish the need to actually model and control for the confounding factors (in addition to input). Which coefficients are fit to
these covariates in the various data sets? How consistent are these coefficients between replicates or between different data sets? How do they depend on different variants of the protocol? How would removing them (all, one, or a subset) affect the results?
d. How is Folding Free Energy evaluated?
e. Parameterization and other choices:
(1) How does changing the bin size affect the model results?
(2)      How is t_min, used to filter regions with insufficient input coverage, selected? Should this value be adjusted according to certain data characteristics? What effect would changing this value have on the model performance?
(3)     Genomic bins with low input coverage are excluded from the model fitting stage, but it is not clear if they are also excluded from the testing stage, and if so - why. Regions with low input coverage could in principle still have high output coverage.

4. Figure 5:
a. The terms "enrichment" (Y-axis in figure 5) is not defined anywhere in the text, as far as
we could tell.
b. Please add the K562 dataset
c. As the authors stated, STARR-seq tests activity regardless of chromatin accessibility. It is,
therefore, possible for closed-chromatin regions to still have regulatory function detected in STARR-seq even if those regions are not active in vivo. While this is certainly a sensible effect to see, the authors should comment on this point.

5. In Figure 6, the authors aim to demonstrate that STARR-Peaker finds more reproducible enhancers. This section suffers from several issues:
a. The datasets used seem to vary greatly in terms of how many peaks overlapped in each one. For example, JKlein2015_MPRA has less than 25 overlapping peaks, whereas FANTOM5_CAGE has more than 2000. The authors should demonstrate that the different datasets indeed find some consensus set of enhancers since these are assumed to be reproducible enhancers.
b. Is the Y-axis fraction or percent? Currently, the figure suggests the maximal overlap is 0.4%, which is extremely low (note that this question cannot be immediately resolved using table 2).
c. For the RatherP2015_STARR-seq dataset, the authors should call peaks for this dataset directly with STARR-Peaker and the other methods and compare the results to the originally called peaks as well as to each other (see the first comment).
d. The rationale for subsampling the peaks to randomly selected 100,000 peaks is not clear. If one method finds significantly more peaks than another, that should be reflected in the analysis and visualization.
e. As mentioned above, if overlaps are computed for each replicate, the authors should note what is the overlap between the replicates themselves.

6. The performance of the method should be evaluated further:
a. Consistency across replicates: how much overlap is there between called peaks across
the two replicates of the same data? How does this compare to other methods?
b. How robust is the method to the quality of the data? The authors could demonstrate
this by subsampling reads at various rates (from either input, output, or both libraries).
c. The authors should simulate STARRSeq data and evaluate their method using data for
which a ground-truth exists. This will help get an idea of the actual sensitivity and
specificity of the method and compare to others.
d. I am missing a comparison of the sets of regions called significant between the various
methods. To what extent do the methods agree? (qualitatively [classification] and
quantitatively [evaluation of signal strength])
e. Competing methods are used with default parameters only. However, since the
characteristics of the datasets used in this manuscript are not necessarily global, it is possible that fine-tuning the parameters of the competing methods could greatly improve performance. The authors should demonstrate that simply fine-tuning competing methods is insufficient to achieve comparable results.


Minor comments:

1. The authors mention binning the genome to 500bp windows in 100bp steps (L133), but later state that the model uses non-overlapping windows. This should be clarified and explained.
2. In the data generated by the authors, peaks are called for each biological replicate separately. How do the authors suggest combining these signals to a unified set of called peaks?
3. The model essentially controls for one coverage signal normalized by another. Can STARRSeq be used for differential peak-calling for other data modalities (i.e ChIP seq), by using a reference signal to normalize a contrast signal? Can it be used for differential peak calling with STARRSeq?
4. The authors mention negative enrichment, but it is not clear if STARR-seq detects negative (silencing) peaks.
5. The authors mention the need for an "activity score", but it seems that STARR-Peaker's main outputs are p-values, not quantitative scores. This should be clarified.
6. The authors describe the advantages of STARR-seq over MPRA. For fairness, they should mention the complimentary limitations of STARR-seq compared with MPRA.
7. Table S2 is not referenced in the text, and there are missing data regarding the number of overlaps found by each method and the number of peaks in each dataset. Please supply these data.
8. The meaning of "consensus sequence" is not clear in the context (L65)
9. The authors use "output library coverage" and "STARR-seq coverage" to refer to the transcribed
RNA library. STARR-seq is both the input and output libraries, so this terminology is confusing.
10. The authors state that they see evidence of negative enrichment (L205). This should be
examined further. Does STARR-Peaker detect these peaks as active peaks as well? Can they be validated?
11. Using NB2 to refer to the negative binomial is not explained, and it cannot be assumed that most readers are aware that there are two forms of the NB distribution. This should be explained and cited, or replaced with a simple "NB".
12. Table S3 is missing accessions for the TF ChIP-seq used to generate Figure 5D
13. The authors describe an alternative mode of operation of their method, "mode 2", but do not
explain when this is useful for users to choose
14. Related to that, it is somewhat confusing to see a fractional observation modeled with a negative binomial (line 432). Please extend the discussion to explain this.

**Authors’ response to reviewers**

REF1.0 – Overall comments

| Referee Comment | Reviewer #1: In this study, Lee and col. developed a bioinformatic pipeline (STARRPeaker) to identify enhancers regions based on high-throughput reporter assays, namely STARR-seq approaches. The processing and analyses of STARR-seq data are generally difficult tasks because the results can be confounded by several potential biases which are not fully addressed by current pipelines. Given the recent interest in high-throughput reporter assays, the development of an accurate pipeline to identify bona fide enhancers is of major interest in the field of functional genomics. Moreover this study also provided whole genome STARR-seq experiments in two human cell lines which are used to validate the STARRPeaker method, but can also represent a valuable resource if properly validated. |
| --- | --- |
| Author Response | We thank the referee for the positive comments and enthusiasm toward this study. In this revision, we have thoroughly addressed the referee’s comments and have made several key improvements over the original submission, as follows:   1. A redrawing of Figure 1, including an analysis of global effects on the alternative fragment counting scheme 2. An additional comparison of STARRPeaker enhancers to known positive controls from other enhancer assays 3. Application of STARRPeaker to external STARR-seq datasets, including captured STARR- seq data 4. Addition of an orientation bias analysis 5. Performance evaluation using subsampling and a simulated control dataset 6. Supporting evidence for model selection 7. Further optimization of the STARR-seq processing pipeline and software parallelization |

REF1.1 – Orientation-dependent biases

| Referee Comment | 1. The STARRPeaker model introduced several covariates to adjust the STARR-seq signal to account for potential biases, including fragment coverage of input genomic libraries, GC content, mappability, and the thermodynamic stability of genomic libraries. Another potential bias might come from the relative orientation of the fragments. Thus, the authors should explore the orientation consistency of their datasets and eventually adjust their method to account for potential orientation-dependent biases. |
| --- | --- |
| Author Response | We agree with the referee that the orientation-dependent activities of the enhancers are of general interest. In the revised manuscript, we have added a new section on orientation bias analysis (Excerpt 1.1.1 and 1.1.2). In summary, we conclude that there is insufficient evidence to establish the presence of orientation bias in our STARR-seq data. |
| Excerpt 1.1.1 (main text) | “… |
|  | *Evaluation of potential orientation bias in candidate enhancers* |
|  | In general, enhancers are thought to function independent of orientation [40]. However, the fragment counts in one orientation could be skewed over the other due to orientation-specific activities, PCR, or sequencing artifacts. To test for potential orientation-based biases, we ran a binomial test on the candidate enhancers we identified. We observed a small fraction of candidate enhancers showing strand bias [3.19% for HepG2 rep1 (n=1,605); 3.76% for HepG2 rep2 (n=1,991); 7.77% for K562 rep1 (n=2,347); 5.25% for K562 rep2 (n=2,195); FDR ≤ 0.01] (Supplementary Figure 5). Less than one third of the enhancers (n=690) showed strand-specific activity in both replicates. Thus, we conclude that there is insufficient evidence to show that orientation-dependent biases are present in our STARR- seq data. Furthermore, this finding provides further support that enhancers function independent of orientation. |
|  | …” |

| Excerpt 1.1.2  (supp. fig. 5) |  |
| --- | --- |

REF1.2 – Correlation between replicates

| Referee Comment | 2. STARR-seq experiments were performed in duplicate. The overall correlation between the replicates should be shown. Besides, the overlap between the enhancers identified in each replicate should be mentioned. |
| --- | --- |
| Author Response | We thank the referee for this suggestion. In the revised manuscript, we evaluate the correlation between two replicates and report a set of reproducible enhancers (Excerpt 1.2.1). In summary, we found a high correlation between two biological replicates (Excerpt 1.2.2). |
| Excerpt 1.2.1 (main text) | “… |
|  | *Initial evaluation of STARRPeaker enhancers* |
|  | We processed two biological replicates from each cell type independently and assessed the correlation between each pair. Overall, we observed high correlation between two replicates (PCC=0.99 for both HepG2 and K562; see Supplementary Figure 3). By intersecting peaks from two replicates, we identified 32,929 and 20,471 reproducible candidate enhancers from HepG2 and K562, respectively (Supplementary Table S1). |
|  | …” |

| Excerpt 1.2.2  (supp. fig. 3) |  |
| --- | --- |

REF1.3 – Comparison to previous WHG-STARR-seq

| Referee Comment | 3. It will be interesting to assess whether similar findings can be observed using other published whole-genome STARR-seq in mammals (e.g., doi:10.1038/nmeth.4534; DOI 10.1186/s13059-017-1345-5). |
| --- | --- |
| Author Response | We thank the referee for this comment. As suggested, the revised manuscript includes a new section presenting the application of our method to other published STARR-seq datasets. Specifically, we applied STARRPeaker to a whole-genome STARR-seq dataset for HeLa-S3 [2] (Excerpt 1.3.1 and 1.3.2). Overall, our results demonstrate that STARRPeaker can be generally applied to other STARR-seq datasets. |
| Excerpt 1.3.1 (main text) | “… |
|  | *Application to external STARR-seq datasets* |
|  | To ensure that STARRPeaker can be generally applied to different variants of STARR-seq assays, we tested STARRPeaker on previously published STARR-seq datasets. |
|  | First, we applied STARRPeaker to the whole-genome ORI-STARR-seq dataset on HeLa-S3 [39] and assessed the quality of the peaks identified. Consistent with the previous claim that IFN-I signaling may induce false-positive enhancers, we identified more peaks in untreated HeLa-S3 samples (n=28,381) compared to inhibitor-treated samples (n=16,150). Furthermore, peaks from untreated samples had lower enrichment of chromatin accessibility (DNase-seq) than those from inhibitor-treated samples, supporting that TBK1/IKK/PKR inhibition reduces false-positive enhancer signals related to IFN-I signaling (Supplementary Figure 9A). Moreover, STARRPeaker covered 77.5% (n=7,451) of published peaks, which were called using BasicSTARRseq and then further shortlisted using a stringent threshold (P-value 1E-5 with corrected enrichment ≥ 4). Furthermore, STARRPeaker found 6,540 additional peaks from a HeLa-S3 sample that was highly enriched with chromatin accessibility signals (Supplementary Figure 9B). |
|  | …” |
| Excerpt 1.3.2  (supp. fig. 9) |  |

REF1.4 – Comparison to known positives

| Referee Comment | 4. To better assess the quality of the resources, the authors should provide several examples of loci comparing the STARR-seq signals and peaks with epigenomic profiles. Whether known enhancers (i.e. independently validated from the literature) are also identified by STARRPeaker should be addressed. For instance, are the enhancers recently identified by CRISPRi in k562 (doi:10.1126/science.aag2445.) identified by the STARRPeaker? |
| --- | --- |
| Author Response | As suggested by the referee, we added a new analysis comparing STARRPeaker enhancers with previously characterized enhancers using CRISPRi in the revised manuscript (Excerpt 1.4.1 and 1.4.2). |
| Excerpt 1.4.1 (main text) | “… |
|  | Second, we examined the nine distal enhancers from the GATA1 and MYC loci characterized in-depth by CRISPRi tiling screen (Supplementary Figure 8). We found that STARRPeaker accurately called peaks for 6 of 9 enhancers from both replicates. For the remaining three regions, we observed insufficient enrichment of STARR-seq output and, therefore, we concluded that this is not a shortcoming of the peak caller. |
|  | …” |
| Excerpt 1.4.2  (supp. fig. 8) |  |

REF1.5 – Enhancers in closed chromatin

| Referee Comment | 5. Previous whole-genome STARR-seq experiments in mammals have suggested that a substantial number of active enhancers are found in closed chromatin (doi:10.1038/nmeth.4534; DOI 10.1186/s13059-017-1345-5). Whether this observation is related to false-positive rate of the used pipelines is not clear. Therefore, what is the percentage of active enhancers found in closed chromatin when using the STARRPeaker |
| --- | --- |

|  | method? Whether the results are consistent or not with the previous finding should be discussed. |
| --- | --- |
| Author Response | As suggested by the referee, we added a discussion on poised enhancers found within closed chromatin in the revised manuscript (Excerpt 1.5). In summary, our results are consistent with previous findings [2,3]. |
| Excerpt 1.5 | “… |
| (main text) | *Initial evaluation of STARRPeaker enhancers* |
|  | We processed two biological replicates from each cell type independently and assessed the correlation between each pair. Overall, we |
|  | observed high correlation between two replicates (PCC=0.99 for both HepG2 and K562; see Supplementary Figure 3). By intersecting |
|  | peaks from two replicates, we identified 32,929 and 20,471 reproducible candidate enhancers from HepG2 and K562, respectively |
|  | (Supplementary Table S1). Although the total number of peaks varied between HepG2 and K562, we observed a comparable number of |
|  | peaks within the accessible region of the genome. We found 12,019 (36.34%) and 11,420 (55.57%) candidate enhancers from HepG2 |
|  | and K562, respectively, within the open chromatin defined by ENCODE DNase-seq hotspots. Consistent with previous findings [39], a |
|  | substantial fraction of candidate enhancers was epigenetically silenced at the chromatin level. However, as demonstrated previously |
|  | using a histone deacetylase inhibitor (HDAC) [16], these poised enhancers can become functional under a more transcriptionally |
|  | permissive environment. Therefore, episomal reporter assays like STARR-seq have the unique advantage of detecting potential |
|  | enhancer activity independent from chromatin context. We would like to note that it is important to identify poised enhancers located in |
|  | heterochromatic regions of the genome, which could become functional during developmental or pathological time courses. |
|  | …” |

REF1.6 – Performance evaluation on captured STARR-seq

| Referee Comment | 6. Many STARR-seq based approaches have been performed with pre-enriched target sequences (e.g. doi: 10.1038/s41467-018-07746-1, doi: 10.1016/j.stem.2018.06.014, DOI: 10.1038/ncomms7905). How the STARRPeaker performs with pre-enriched based reporter assays is an important issue, as these targeted approaches might become very popular. |
| --- | --- |
| Author Response | We thank the referee for this comment. In the revised manuscript, we apply STARRPeaker to a captured STARR-seq dataset and evaluate its performance (Excerpt 1.6). |
| Excerpt 1.6 (main text) | “… |
|  | Second, we tested if STARRPeaker can be reliably applied to captured STARR-seq datasets (Cap-STARR-seq). We applied STARRPeaker to a previously characterized GM12878 STARR-seq dataset based on an ATAC-seq-capture technique called HiDRA  [43] and compared its performance with published results. The HiDRA dataset was reported to have ~65,000 regions with enhancer function. In the STARRPeaker run, we identified only 20,852 regions with significant enhancer activities from the five replicates they produced. Approximately 73.6% of peaks overlapped with the published results (n=15,347). While it is debatable to claim that one method is superior to the other, this result demonstrates that STARRPeaker can be reliably used against the Cap-STARR-seq dataset. |
|  | …” |

REF1.7 – List of enhancers

| Referee Comment | 7. A table listing the enhancers identified from each experiment should be provided. |
| --- | --- |
| Author Response | We provided a list of ORI-STARR-seq enhancers identified by STARRPeaker in Supplementary Table 1. In addition, users can download all raw and derived resources from the ENCODE portal (ENCSR135NXN for HepG2 and ENCSR858MPS for K562). |

REF1.8 – Typo

| Referee Comment | 8. Typo: "We also filtered out read pairs that had a fragment insert size less than and greater than.": It should be the opposite |
| --- | --- |
| Author Response | We appreciate the referee’s attentiveness in catching typos. This mistake has been fixed; furthermore, we have ensured that the revised manuscript is free of typos and grammatical errors. |
| Excerpt 1.7 (main text) | “…We also filtered out read pairs that had a fragment insert size greater than 𝑙_"#$_ and less than 𝑙_"%&_. By default, we filtered out fragment insert sizes less than 200 bp and greater than 1,000 bp. …” |

REF2.0 – Overall comments

| Referee Comment | Reviewer #2: Lee et al. introduce STARR-Peaker, a peak-calling algorithm designed specifically to analyze STARR-seq data, an increasingly popular method for detecting active enhancer sequences. The authors demonstrate their approach using two STARR-seq datasets generated in this study, and compare STARR-Peaker with several existing methods. While a method designed for STARR-seq may be of interest to the Genome Biology readership, we feel that the authors do not present sufficiently convincing evidence of the superiority and applicability of their approach. Several aspects of the method and analyses are not clearly explained and the model is not sufficiently evaluated both in terms of the datasets used and the performance analyses |
| --- | --- |
| Author Response | We thank the referee for the positive comments and enthusiasm toward this study. In this revision, we have fully addressed the referee’s comments and have made several key improvements over the original submission, including the following:   1. A redrawing of Figure 1, including an analysis of global effects on the alternative fragment counting scheme. 2. An additional comparison of STARRPeaker enhancers with known positive controls from other enhancer assays 3. Application of STARRPeaker to external STARR-seq datasets, including captured STARR- seq data 4. Addition of an orientation bias analysis 5. Performance evaluation using subsampling and a simulated control dataset 6. Supporting evidence for model selection 7. Further optimization of the STARR-seq processing pipeline and software parallelization |

REF2.1 – External datasets

| Referee Comment | 1. Data: The authors perform all evaluations of STARR-seq properties and of their method using two datasets generated by the authors using the same protocol. To demonstrate that the biases are general and the method is generally applicable and superior, additional external datasets should be used. |
| --- | --- |
| Author Response | We thank the referee for this constructive suggestion. In the revised manuscript, we have included extensive comparisons and applications to various external datasets (K562 Cap-STARRR-seq from Rathert et al. 2015, K562 CRISPRi from Fulco et al. 2016, HeLa-S3 ORI-STARR-seq from Muerdter et al. 2018, and GM12878 ATAC-STARR-seq from Wang et al. 2018). |
| Excerpt 2.1 (main text) | “…  *Comparison to previously characterized enhancers*  First, we compared the peaks identified by STARRPeaker to previously characterized enhancers from HepG2 or K562 cell lines by CAGE [41], MPRA [17,42], and STARR-seq [19] (Figure 6, Supplementary Table S2. …  Second, we examined the nine distal enhancers from the GATA1 and MYC loci characterized in-depth by CRISPRi tiling screen (Supplementary Figure 8). …  *Application to external STARR-seq datasets*  To ensure that STARRPeaker can be generally applied to different variants of STARR-seq assays, we tested STARRPeaker on previously published STARR-seq datasets. |

|  | First, we applied STARRPeaker to the whole-genome ORI-STARR-seq dataset on HeLa-S3 [39] and assessed the quality of the peaks identified. …  Second, we tested if STARRPeaker can be reliably applied to captured STARR-seq datasets (Cap-STARR-seq). We applied STARRPeaker to a previously characterized GM12878 STARR-seq dataset based on an ATAC-seq-capture technique called HiDRA  [43] and compared its performance with published results. …  Third, we further evaluated the performance of the peak-calling methods by applying STARRPeaker and two other peak-calling methods to another published Cap-STARR-seq dataset [19]. …  …” |
| --- | --- |

REF2.2.1 – Figure 1: fragment counting scheme

| Referee Comment | 2. Figure 1:  a. The figure aims to demonstrate the effect of using the fragment center as opposed to the beginning to associate reads to bins. However, the authors do not establish that this decision is beneficial, only that it potentially changes the peak centers. Even if the change is significant (see below) - it should be demonstrated that it results in more accurate peaks positioning. This can be done, for instance, by running a variant of the model with alternative fragment counting schemes. |
| --- | --- |
| Author Response | We thank the referee for this comment. As suggested, we have carefully assessed the benefit of using fragment-based coverage in the revised manuscript (Excerpt 2.2.1). In summary, the fragment-based coverage offers more concentrated and robust peak signals than an alternative coverage counting scheme. |
| Excerpt 2.2.1 (main text) | “… |
|  | To assess the benefit of using fragment-based coverage, we compared the coverage calculated using the center of fragment insert to an alternate model using the start position of the sequencing read. We found that the position of the peaks shifted up approximately 200 bp when we used the alternate model (Figure 1A, Supplementary Figure 1A). Such a shift caused by the read-based coverage could lead to the omission of TF binding sites located at the boundary. Moreover, we observed that the read-based coverage diluted the overall STARR-seq signal; as a result, peaks calculated based on the alternate model had lower fold enrichment and were less confident and broader in size (Figure 1B-D, Supplementary Figure 1B-D). Overall, the fragment-based coverage offered more concentrated and robust peak signal compared to the read-based coverage counting scheme. The benefit of using the center of the fragment is highlighted in Figure 1E, where we find more concise and precise peak with a higher fold enrichment using fragment-based coverage. |
|  | …” |

REF2.2.2 – Figure 1: global assessment of fragment counting scheme

| Referee Comment | b. The figure shows the effect on four regions, but neither the figure nor the text quantify this effect across all regions or make any statements about how consistent or significant it is. |
| --- | --- |
| Author Response | We have redrawn Figure 1, which now includes global statistics regarding the benefit (Excerpt 2.2.2). |

| Excerpt 2.2.2  (main fig. 1) |  |
| --- | --- |

REF2.2.3 – Figure 1: bin size

| Referee Comment | c. The figure uses a bin size of 50bp, but throughout the paper the authors use 500bp. Please clarify. |
| --- | --- |
| Author Response | We did in fact use a 500 bp bin with a 100 bp sliding window in the original figure. However, this issue is no longer relevant, as we have redrawn the figure (Excerpt 2.2.2). |

REF2.2.4 – Figure 1: shift in peak

| Referee Comment | d. When examining the regions selected, it appears that the shift in the peak center falls within the same 500bp window in all cases, which seems to mean that this shift won't affect any of the downstream analyses. Please comment on how this decision affects the results of the model. |
| --- | --- |
| Author Response | We have expanded our analysis for comparison with an alternate coverage counting scheme in the revised manuscript (Excerpt 2.2.1). |

REF2.3.1 – Figure 2: null model

| Referee Comment | 3. Model and Figure 2: |
| --- | --- |

|  | a. The model description should be clarified and simplified. It is not immediately clear from the text that STARR-Peaker fits a single null model across all genomic bins, and then tests each genomic bin using the null model to compute p-values. Related to that - there seems to be a typo in the p-value equation (line 287). |
| --- | --- |
| Author Response | We thank the referee for identifying the typo in the equation. In the revised manuscript, we have simplified and clarified the model description. We have also rewritten the section on the null model (Excerpt 2.3.1) and on the p-value computation (Excerpt 2.3.2). |
| Excerpt 2.3.1 (main text) | “…  Our model starts by fitting an analytical distribution to the observed fragment coverage across fixed non-overlapping genomic bins. In doing so, we use covariates to model expected counts in the form of multiple regression. Subsequently, once a model is fitted, we evaluate the likelihood of obtaining the observed fragment counts and assign p-values using the null negative binomial distribution. In this testing phase, we use flexible genomic bins with a sliding window in order to find enrichment peaks at a higher resolution.  Genomic bins with significant enrichments are selected based on their adjusted p-values using multiple testing correction. Finally, peak locations are fine-tuned to the summit of the direct fragment coverage. Note that the adjusted p-value should be regarded as the likelihood of a candidate region being an enhancer while the fold enrichment can be directly interpreted as a quantitative measure of enhancer activity.  …  *Estimation of P-value*  The P-value is defined as the probability of observing equal or more extreme value than the observed value at the 𝑖-th bin, 𝑦_%_, under the null hypothesis.  𝑃– 𝑣𝑎𝑙𝑢𝑒_%_ = Pr(𝑌 ≥ 𝑦_%_\|𝐻) (7)  As defined earlier, we assume the random variable Y comes from a negative binomial distribution with the fitted mean at the 𝑖-th bin,  𝜇_%_, as the expected value, and 𝜃 as the dispersion parameter. Then, we can estimate the P-value from the cumulative distribution function 𝐶𝐷𝐹, which is the sum of the probability mass function 𝑓_>_ from 0 to 𝑦_%_ − 1.  DEFG  Pr(𝑌 ≥ 𝑦_%_\|𝐻) = 1 − 𝐶𝐷𝐹(𝑦_%_ − 1) = 1 − A 𝑓_>_(𝑘\|𝜇_%_, 𝜃) (8)  HIJ  Substituting (1) gives  DEFG  Γ(𝑘 + 𝜃) 𝜃 ^P^ 𝜇_%_ H  𝑃– 𝑣𝑎𝑙𝑢𝑒_%_ = 1 − A Γ(𝑘 + 1) ∙ Γ(𝜃) ∙ N𝜃 + 𝜇 O ∙ N𝜃 + 𝜇 O (1)  HIJ % %  Finally, we calculate the false discovery rate using Benjamini & Hochberg method [38].  …” |

REF2.3.2 – Figure 2: DNA term and other biases

| Referee Comment | b. From Figure 2 it looks like the DNA and RNA counts are highly correlated. It may, therefore, be the case that some of the other biases (e.g., GC content, and maybe |
| --- | --- |

|  | confusingly enough - RNA structure properties) also confound the DNA data. This means that in the model, it -may- be sufficient to regress only the DNA term. The authors should explore this point and also report the confounding of the DNA. |
| --- | --- |
| Author Response | As noted by the referee, some of the surveyed covariates are sequence based and therefore may potentially be correlated. However, this possibility does not imply that the other terms do not contribute to the model. In the revised manuscript, we acknowledge this point and explore an alternate model without covariates. |
| Excerpt 2.3.2 (main text) | “… |
|  | Based on these findings, we built a regression-based model that accounts for various confounding variables of test sequence fragments to unbiasedly identify potential enhancer regions from STARR-seq data. Note that many of the covariates have appreciable correlation with each other. However, we did find, using stepwise forward selection, that each of them contributes substantially and independently to the model fit as assessed by Akaike information criterion (AIC) and Bayesian information criterion (BIC) (Supplementary Figure 2). |
|  | …” |

REF2.3.3 – Figure 2: covariates

| Referee Comment | c. More generally, the authors should better establish the need to actually model and control for the confounding factors (in addition to input). Which coefficients are fit to these covariates in the various data sets? How consistent are these coefficients between replicates or between different data sets? How do they depend on different variants of the protocol? How would removing them (all, one, or a subset) affect the results? |
| --- | --- |
| Author Response | As suggested by the referee, we have quantified the model selection process in the revised manuscript (Excerpt 2.3.2 and 2.3.3). |

| Excerpt 2.3.3  (suppl. fig. 2) |  |
| --- | --- |

REF2.3.4 – Figure 2: folding free energy

| Referee Comment | d. How is Folding Free Energy evaluated? |
| --- | --- |
| Author Response | We have added a new method section describing the calculation of the folding free energy for each genomic bin (Excerpt 2.3.4). |
| Excerpt 2.3.4 (main text) | “… |
|  | **Calculating folding free energy** |
|  | We used the LinearFold [46] algorithm to estimate the folding energy of each genomic bin iteratively across the whole genome. Specifically, we used the Vienna RNAfold thermodynamic model [47] with parameters from Mathews et al. 2004 [48]. We implemented a parallel processing scheme to leverage multicore processors to expedite the calculation of folding free energy. |
|  | …” |

REF2.3.5 – Figure 2: parameterization

| Referee Comment | e. Parameterization and other choices:   (1) How does changing the bin size affect the model results? (2) How is t_min, used to filter regions with insufficient input coverage, selected? Should this value be adjusted according to certain data characteristics? What effect would changing this value have on the model performance? |
| --- | --- |

|  | (3) Genomic bins with low input coverage are excluded from the model fitting stage, but it is not clear if they are also excluded from the testing stage, and if so - why. Regions with low input coverage could in principle still have high output coverage. |
| --- | --- |
| Author Response | We thank the referee for these suggestions. We have clarified these points in the revised manuscript (Excerpt 2.3.5). In summary, the bin size and minimum coverage were determined based on the STARR-seq experimental design. Genomic bins with low coverage were excluded from both model fitting and testing. |
| Excerpt 2.3.5 (main text) | “… |
|  | Therefore, we only considered a non-overlapping subset of bins for modeling and fitting the distribution. We also excluded bins not covered by any genomic input or those in which the normalized input coverage was less than a minimum quantile t_min, since these regions do not have sufficient power to detect enrichment. We selected the bin size and the minimum coverage based on the experimental design of STARR-seq. |
|  | …” |

REF2.4.1 – Figure 5: caption

| Referee Comment | 4. Figure 5:  a. The terms "enrichment" (Y-axis in figure 5) is not defined anywhere in the text, as far as we could tell. |
| --- | --- |
| Author Response | We thank the referee for identifying this oversight. We have revised the caption for Figure 5 in the revised manuscript (Excerpt 2.4.1). |
| Excerpt 2.4.1 (main text) | “… |
|  | **Figure 5** Enrichment of epigenetic signals around peaks in HepG2. All peaks were centered at the summit, uniformly thresholded using P-value < 0.001, and 10,000 peaks were randomly selected. Aggregated read depth at 2,000 bp upstream and downstream were plotted for **(A)** DNase I hypersensitive sites (DHS), **(B)** H3K27ac, **(C)** H3K4me1, and **(D)** aggregated TF ChIP-seq profile. For DNase-seq, enrichment indicates unique read depth. For histone ChIP-seq, enrichment indicates fold change over control. For TF ChIP-seq aggregate, enrichment indicates the number of TFs binding. |
|  | …” |

REF2.4.2 – Figure 5: K562 dataset

| Referee Comment | b. Please add the K562 dataset |
| --- | --- |
| Author Response | We have added the K562 dataset as Supplementary Figure 7 (Excerpt 2.4.2). |

| Excerpt 2.4.2  (supp. fig. 7) |  |
| --- | --- |

REF2.4.3 – Figure 5: closed chromatin region

| Referee Comment | c. As the authors stated, STARR-seq tests activity regardless of chromatin accessibility. It is, therefore, possible for closed-chromatin regions to still have regulatory function detected in STARR-seq even if those regions are not active in vivo. While this is certainly a sensible effect to see, the authors should comment on this point. |
| --- | --- |
| Author Response | As suggested by the referee, we have added a discussion on poised enhancers found within closed chromatin in the revised manuscript (Excerpt 2.4.3). In summary, we view this point as a strength of the STARR-seq assay, as these endogenously repressed regions should still be regarded as developmentally or pathologically functional. |
| Excerpt 2.4.3 (main text) | “…  *Initial evaluation of STARRPeaker enhancers*  We processed two biological replicates from each cell type independently and assessed the correlation between each pair. Overall, we observed high correlation between two replicates (PCC=0.99 for both HepG2 and K562; see Supplementary Figure 3). By intersecting peaks from two replicates, we identified 32,929 and 20,471 reproducible candidate enhancers from HepG2 and K562, respectively (Supplementary Table S1). Although the total number of peaks varied between HepG2 and K562, we observed a comparable number of peaks within the accessible region of the genome. We found 12,019 (36.34%) and 11,420 (55.57%) candidate enhancers from HepG2 and K562, respectively, within the open chromatin defined by ENCODE DNase-seq hotspots. Consistent with previous findings [39], a substantial fraction of candidate enhancers was epigenetically silenced at the chromatin level. However, as demonstrated previously using a histone deacetylase inhibitor (HDAC) [16], these poised enhancers can become functional under a more transcriptionally permissive environment. Therefore, episomal reporter assays like STARR-seq have the unique advantage of detecting potential enhancer activity independent from chromatin context. We would like to note that it is important to identify poised enhancers located in heterochromatic regions of the genome, which could become functional during developmental or pathological time courses. |
|  | …” |

REF2.5.1 – Figure 6:

| Referee Comment | 5. In Figure 6, the authors aim to demonstrate that STARR-Peaker finds more reproducible enhancers. This section suffers from several issues:    a. The datasets used seem to vary greatly in terms of how many peaks overlapped in each one. For example, JKlein2015_MPRA has less than 25 overlapping peaks, whereas FANTOM5_CAGE has more than 2000. The authors should demonstrate that the different datasets indeed find some consensus set of enhancers since these are assumed to be reproducible enhancers. |
| --- | --- |

| Author Response | The majority of the published datasets utilized in this work were not derived from whole-genome- scale assays. For example, the STARR-seq dataset from Rathert et al. 2015 covers only the 3.1 MB surrounding regions of the MYC locus, while the MRPA dataset from Klein et al. 2019 tested only 62 regions. Furthermore, the FANTOM5 CAGE dataset has 63,285 identified enhancers based on the CAGE transcription initiation signal. These datasets are suboptimal but are currently the best resources available for performance evaluation. We clarified this point and strengthened our comparison in the revised manuscript. Due to the varying scopes of the other datasets, we did not designate them as a consensus set of enhancers; instead, we published a detailed summary of the comparison with external datasets in Supplementary Table S2. |
| --- | --- |
| Excerpt 2.5.1 | “… |
| (main text) | *Comparison to previously characterized enhancers* |
|  | First, we compared the peaks identified by STARRPeaker to previously characterized enhancers from HepG2 or K562 cell lines by |
|  | CAGE [41], MPRA [17,42], and STARR-seq [19] (Figure 6, Supplementary Table S2). Overall, we observed a higher fraction of |
|  | STARRPeaker peaks overlapping with external datasets compare to other methods. Moreover, we found higher overlaps when peaks |
|  | from both replicates were merged, due to fewer but more precise candidate enhancers from merging replicates. However, we noticed |
|  | reduced agreement across different types of enhancer assays. Low overlap between assays may arise from different formats or layouts |
|  | of reporter plasmids, such as differing enhancer cloning sites or promoters, or differences in the complexity of the screening library. |
|  | Furthermore, CAGE is an entirely different assay from episomal reporter assays like MPRA and STARR-seq, with enhancers defined |
|  | based on bidirectional transcripts originating from an eRNA. |
|  | Second, we examined the nine distal enhancers from the GATA1 and MYC loci characterized in-depth by CRISPRi tiling screen |
|  | (Supplementary Figure 8). We found that STARRPeaker accurately called peaks for 6 of 9 enhancers from both replicates. For the |
|  | remaining three regions, we observed insufficient enrichment of STARR-seq output and, therefore, we concluded that this is not a |
|  | shortcoming of the peak caller. |
|  | …” |

REF2.5.2 – Figure 6: label

| Referee Comment | b. Is the Y-axis fraction or percent? Currently, the figure suggests the maximal overlap is 0.4%, which is extremely low (note that this question cannot be immediately resolved using table 2). |
| --- | --- |
| Author Response | We thank the referee for identifying this error. The y-axis in Figure 6 was incorrectly labeled as a fraction in the original submission. We have revised the y-axis label in this figure to reflect the percent overlap (Excerpt 2.5.2). |
| Excerpt 2.5.2  (main fig. 6) |  |

REF2.5.3 – Figure 6: external dataset

| Referee Comment | c. For the RatherP2015_STARR-seq dataset, the authors should call peaks for this dataset directly with STARR-Peaker and the other methods and compare the results to the originally called peaks as well as to each other (see the first comment). |
| --- | --- |

| Author Response | As suggested by the referee, we directly call peaks from the dataset of Rathert et al. 2015 and compare the results in the revised manuscript (Excerpt 2.5.3). |
| --- | --- |
| Excerpt 2.5.3 (main text) | “… |
|  | Third, we further evaluated the performance of the peak-calling methods by applying STARRPeaker and two other peak-calling methods to another published Cap-STARR-seq dataset [19]. The dataset covers approximately 91% of the surrounding 3 Mb of the MYC locus. Consistent with the earlier analysis, we observed that STARRPeaker is highly specific and identifies fewer candidate enhancers (n=26) compared to the other methods (BasicSTARRseq n=223; MACS2 n=136). Furthermore, a four-way comparison (STARRPeaker, BasicSTARRseq, MACS2, and published peaks) showed that all of the STARRPeaker peaks overlapped with peaks from other methods but not the other way around (Supplementary Figure 10). These results indicate that STARRPeaker is more robust and reliable at identifying reproducible candidate enhancers from various STARR-seq datasets than previous methods. |
|  | …” |

REF2.5.4 – Figure 6: subsampling

| Referee Comment | d. The rationale for subsampling the peaks to randomly selected 100,000 peaks is not clear. If one method finds significantly more peaks than another, that should be reflected in the analysis and visualization. |
| --- | --- |
| Author Response | In the revised manuscript, we have clarified that other methods find more peaks and have added our rationale for subsampling peaks in the performance comparison (Excerpt 2.5.4). |
| Excerpt 2.5.4 (main text) | “… |
|  | While it is uncertain how many true enhancers were present in each sample, we had to ensure that we made a fair comparison across different methods due to the tradeoff between sensitivity and specificity. An increase in sensitivity is generally achieved at the expense of a decrease in specificity, as described in receiver operating characteristic curves. In our context, a method having higher specificity suffers from having less overlap with open chromatin and previously identified enhancers from other assays. Therefore, we used a uniform P-value threshold of 0.001 and subsampled the peaks before the comparison. |
|  | …” |

REF2.5.5 – Figure 6: overlap between replicate-merged peaks

| Referee Comment | e. As mentioned above, if overlaps are computed for each replicate, the authors should note what is the overlap between the replicates themselves. |
| --- | --- |
| Author Response | Based on the referee’s suggestion, we have revised Figure 6 (Excerpt 2.5.2). We have also added a comparison with peaks merged from both replicates in the revised manuscript (Excerpt 2.5.1). |

REF2.6.1 – Performance evaluation across replicates

| Referee Comment | 6. The performance of the method should be evaluated further:  a. Consistency across replicates: how much overlap is there between called peaks across the two replicates of the same data? How does this compare to other methods? |
| --- | --- |
| Author Response | In the revised manuscript, we report the number of peak overlaps between replicates (Excerpt 2.6.1). In addition, we compare the number of peaks that are reproducible across different methods in Supplementary Table S4. |

| Excerpt 2.6.1 (main text) | “… |
| --- | --- |
|  | *Initial evaluation of STARRPeaker enhancers* |
|  | We processed two biological replicates from each cell type independently and assessed the correlation between each pair. Overall, we observed high correlation between two replicates (PCC=0.99 for both HepG2 and K562; see Supplementary Figure 3). By intersecting peaks from two replicates, we identified 32,929 and 20,471 reproducible candidate enhancers from HepG2 and K562, respectively (Supplementary Table S1). |
|  | …” |

REF2.6.2 – Performance evaluation

| Referee Comment | b. How robust is the method to the quality of the data? The authors could demonstrate this by subsampling reads at various rates (from either input, output, or both libraries). |
| --- | --- |
| Author Response | As suggested by the referee, we subsampled the STARR-seq library at various rates to evaluate the robustness of this method (Excerpt 2.6.2). |
| Excerpt 2.6.2 (main text) | “… |
|  | *Assessment of robustness and reproducibility of the method* |
|  | A reliable peak-calling method should be able to identify peaks from suboptimal datasets. To evaluate the robustness of STARRPeaker, we used subsets of the whole-genome STARR-seq library to call peaks and compared the results. We subsampled randomly at various rates from 20 to 80% of the total dataset and compared the quality of peaks. We found that STARRPeaker was able to reliably identify the peaks using approximately 60% of the original sequencing library (Supplementary Figure 4). However, the quality of the peak calls started to deteriorate when 40% or less were used. |
|  | …” |
|  |  |

REF2.6.3 – Performance evaluation: simulating data with ground truth

| Referee Comment | c. The authors should simulate STARRSeq data and evaluate their method using data for which a ground-truth exists. This will help get an idea of the actual sensitivity and specificity of the method and compare to others. |
| --- | --- |
| Author Response | We thank the referee for this suggestion. In the revised manuscript, we create a simulated dataset for which a ground truth exists and perform a qualitative assessment of peak-calling algorithms (Excerpt 2.6.3). |
| Excerpt 2.6.3 (main text) | “… |
|  | First, we qualitatively assessed the peak-calling algorithms using a simulated dataset where the ground truth exists. We created a STARR-seq dataset that consists of four spike-in controls (hybrid of DNA input library and RNA output library of known specific location). All three methods successfully identified the four control peaks with high confidence (Supplementary Figure 6). However, we noticed that BasicSTARRseq peaks were fragmented due to its limitation of fixed peak size. Moreover, the peaks were shifted toward the enrichment of sequencing reads. Furthermore, BasicSTARRseq identified a false-positive peak, and as a result, identified a total of eight regions instead of four. |
|  | …” |

REF2.6.4 – Performance evaluation

| Referee Comment | d. I am missing a comparison of the sets of regions called significant between the various methods. To what extent do the methods agree? (qualitatively [classification] and quantitatively [evaluation of signal strength]) |
| --- | --- |
| Author Response | Considering the referee’s suggestion, we have completely rewritten the performance evaluation section. We have strengthened the comparison using simulated, internal, and external STARR-seq datasets and have included both qualitative and quantitative assessments among methods in the revised manuscript (Excerpt 2.6.4). |
| Excerpt 2.6.4 (main text) | “… |
|  | *Performance comparison to other peak-calling algorithms* |
|  | We evaluated the performance of STARRPeaker by comparing it to previously used methods, namely BasicSTARRseq and MACS2. |
|  | First, we qualitatively assessed the peak-calling algorithms using a simulated dataset where the ground truth exists. … |
|  | Second, we quantitatively assessed the peak-calling algorithms using the whole human genome STARR-seq dataset. After uniformly calling peaks from each method using the recommended default settings, we evaluated the quality of the candidate enhancers identified. We found that both BasicSTARRseq and MACS2 called significantly more peaks (4 to 20-fold higher) than STARRPeaker (Supplementary Table S4). While it is uncertain how many true enhancers were present in each sample, we had to ensure that we made a fair comparison across different methods due to the tradeoff between sensitivity and specificity. An increase in sensitivity is generally achieved at the expense of a decrease in specificity, as described in receiver operating characteristic curves. In our context, a method having higher specificity suffers from having less overlap with open chromatin and previously identified enhancers from other assays. Therefore, we used a uniform P-value threshold of 0.001 and subsampled the peaks before the comparison. After uniformly processing the dataset using each method, we measured the level of epigenetic profile enrichment around the peaks. We observed higher enrichment of DNase-hypersensitive sites, as well as more distinct double-peak patterns of H3K27ac and H3K4me1, using STARRPeaker compared to BasicSTARRseq or MACS2 (Figure 5, Supplementary Figure 7). Furthermore, STARRPeaker peaks had significantly higher enrichment of TF binding events (based on the number of TF ChIP-seq binding sites) compared to the peaks identified using other methods. |
|  | …” |

REF2.6.5 – Performance evaluation

| Referee Comment | e. Competing methods are used with default parameters only. However, since the characteristics of the datasets used in this manuscript are not necessarily global, it is possible that fine-tuning the parameters of the competing methods could greatly improve performance. The authors should demonstrate that simply fine-tuning competing methods is insufficient to achieve comparable results. |
| --- | --- |
| Author Response | We thank the referee for this suggestion. We have decided to stay with using the current parameters for several reasons. First, the authors of each program have already carefully fine-tuned the default parameters, Second, some parameters are method-specific, and we find that tuning may alter the designed operation of the program. Finally, the previous works have used default parameters, and unless there is a specific reason for changing them, leaving the parameters at their default values is the only way to conduct a fair comparison. In light of these facts, we consider the exploration of each program's parameters to lie beyond the scope of the current work. |

REF2.7.1 – (Minor): genomic bins

| Referee Comment | Minor comments:  1. The authors mention binning the genome to 500bp windows in 100bp steps (L133), but later state that the model uses non-overlapping windows. This should be clarified and explained. |
| --- | --- |
| Author Response | As suggested by the reviewer, we revised the manuscript to clarify this point (Excerpt 2.7.1). In summary, any discrete probability distribution assumes a sequence of independent and identically distributed Bernoulli trials. Therefore, during the training phase, we used non-overlapping windows to ensure that each genomic bin is independent. During the testing phase, we used sliding windows to identify enrichment peaks at a higher resolution. Both the window size and step size are parametrized and can be adjusted according to the user’s preference. |
| Excerpt 2.7.1 (main text) | “… |
|  | To model the fragment coverage data from STARR-seq using discrete probability distribution, we assumed that each genomic bin is |
|  | independent and identically distributed, as specified in the Bernoulli trials [33]. That is, each test fragment can only map to a single |
|  | fixed-length bin. Therefore, we only considered a non-overlapping subset of bins for modeling and fitting the distribution. |
|  | … |
|  | Our model starts by fitting an analytical distribution to the observed fragment coverage across fixed non-overlapping genomic bins. In |
|  | doing so, we use covariates to model expected counts in the form of multiple regression. Subsequently, once a model is fitted, we |
|  | evaluate the likelihood of obtaining the observed fragment counts and assign p-values using the null negative binomial distribution. In |
|  | this testing phase, we use flexible genomic bins with a sliding window in order to find enrichment peaks at a higher resolution. |
|  | Genomic bins with significant enrichments are selected based on their adjusted p-values using multiple testing correction. Finally, peak |
|  | locations are fine-tuned to the summit of the direct fragment coverage. |
|  | …” |

REF2.7.2 – (Minor): merge replicates

| Referee | Minor comments: |
| --- | --- |
| Comment | 2. In the data generated by the authors, peaks are called for each biological replicate |
|  | separately. How do the authors suggest combining these signals to a unified set of called |
|  | peaks? |

| Author Response | The best practice is to process each replicate independently and then evaluate the reproducibility across replicates. In the revised manuscript, we provide a unified set of peaks merging two replicates in Supplementary Table S1. |
| --- | --- |

REF2.7.3 – (Minor): use for ChIP-seq

| Referee Comment | Minor comments:  3. The model essentially controls for one coverage signal normalized by another. Can STARRSeq be used for differential peak-calling for other data modalities (i.e ChIP seq), by using a reference signal to normalize a contrast signal? Can it be used for differential peak calling with STARRSeq? |
| --- | --- |
| Author Response | In principal, STARRPeaker can be used for other data modalities, such as ChIP-seq; however, we would need to re-evaluate the covariates. We believe such work is beyond the scope of the current study. |

REF2.7.4 – (Minor): negative enrichment

| Referee Comment | Minor comments:  4. The authors mention negative enrichment, but it is not clear if STARR-seq detects negative (silencing) peaks. |
| --- | --- |
| Author Response | In the revised manuscript, we have clarified this point and have enhanced our discussion on negative enrichment of the STARR-seq output coverage (Excerpt 2.7.4). |
| Excerpt 2.7.4 (main text) | “… |
|  | We observed a slight negative enrichment in the STARR-seq output coverage, suggesting that some candidate fragments can repress the basal transcriptional activity. However, these regions may contain sequences that can destabilize mRNAs. Therefore, additional experiments are necessary to demonstrate that STARR-seq can reliably detect silencers. In the meantime, we suggest opting for a system specifically designed for identifying silencers for this task [34]. |
|  | …” |

REF2.7.5 – (Minor): activity score

| Referee Comment | Minor comments:  5. The authors mention the need for an "activity score", but it seems that STARR-Peaker's main outputs are p-values, not quantitative scores. This should be clarified. |
| --- | --- |
| Author Response | We have clarified this point in the revised manuscript (Excerpt 2.7.5). In summary, in addition to the p-value significance of the peak, we report the fold enrichment over the normalized input, which can be directly interpreted as a quantitative activity score. |
| Excerpt 2.7.5 (main text) | “…  Note that the adjusted p-value should be regarded as the likelihood of a candidate region being an enhancer while the fold enrichment can be directly interpreted as a quantitative measure of enhancer activity. |

|  | …” |
| --- | --- |

REF2.7.6 – (Minor): limitation of STARR-seq compared to MPRA

| Referee Comment | Minor comments:  6. The authors describe the advantages of STARR-seq over MPRA. For fairness, they should mention the complimentary limitations of STARR-seq compared with MPRA. |
| --- | --- |
| Author Response | We thank the referee for this comment. We have added the limitations of STARR-seq compared with MPRA in the revised manuscript (Excerpt 2.7.6). |
| Excerpt 2.7.6 (main text) | “… |
|  | Importantly, STARR-seq can be scaled to the whole-genome level for unbiased scanning of functional activities. However, scaling STARR-seq to the human genome is still very challenging, primarily due to its massive size. A more complex genomic DNA library, a higher sequencing depth, and increased transfection efficiency are required to cover the whole human genome [16], which could ultimately introduce biases. Furthermore, inserting a large fragment of DNA into the 3' UTR of the reporter gene could inadvertently introduce regulatory sequences that might affect mRNA abundance and stability, which could lead to both false positives and false negatives. MPRA is more robust in this regard because the activity of each candidate enhancer is quantified by multiple molecular barcodes associated with the fragment, making it less prone to such artifacts than STARR-seq. |
|  | …” |

REF2.7.7 – (Minor): Table S2

| Referee Comment | Minor comments:  7. Table S2 is not referenced in the text, and there are missing data regarding the number of overlaps found by each method and the number of peaks in each dataset. Please supply these data. |
| --- | --- |
| Author Response | In the original submission, two tables, S2-1 and S2-2, were listed as separate tabs within the Supplementary Table S2 file. In the revised manuscript, we divided this table into Supplementary Table S2 and S4. We have also included an additional reference to this table in the revised manuscript (Excerpt 2.7.7). |
| Excerpt 2.7.7 (main text) | “… |
|  | *Comparison to previously characterized enhancers* |
|  | First, we compared the peaks identified by STARRPeaker to previously characterized enhancers from HepG2 or K562 cell lines by CAGE [41], MPRA [17,42], and STARR-seq [19] (Figure 6, Supplementary Table S2). Overall, we observed a higher fraction of STARRPeaker peaks overlapping with external datasets compare to other methods. |
|  | …” |

REF2.7.8 – (Minor): consensus sequence

| Referee Comment | Minor comments:  8. The meaning of "consensus sequence" is not clear in the context (L65) |
| --- | --- |

| Author Response | We revised the manuscript to clarify this term (Excerpt 2.7.8). In summary, we use the term “consensus sequence” to denote a sequence of nucleotides that characterize enhancer function. |
| --- | --- |
| Excerpt 2.7.8 (main text) | “… |
|  | Consensus sequences (or canonical sequences) have been identified at certain protein binding sites, splice sites, and boundaries of protein-coding genes. However, there are no known consensus sequences that characterize enhancer function, making it challenging to identify enhancers based on sequence alone in an unbiased fashion. |
|  | …” |

REF2.7.9 – (Minor): STARR-seq coverage

| Referee Comment | Minor comments:  9. The authors use "output library coverage" and "STARR-seq coverage" to refer to the transcribed RNA library. STARR-seq is both the input and output libraries, so this terminology is confusing. |
| --- | --- |
| Author Response | We thank the referee for this comment. We admit that these terms were loosely defined in the original submission. In the revised manuscript, we have corrected this oversight, explicitly referring to STARR-seq input or output coverage and avoiding the term “STARR-seq coverage” unless referring to both the input and output. |

REF2.7.10 – (Minor): negative enrichment

| Referee Comment | Minor comments:  10. The authors state that they see evidence of negative enrichment (L205). This should be examined further. Does STARR-Peaker detect these peaks as active peaks as well? Can they be validated? |
| --- | --- |
| Author Response | As discussed in REF2.7.4, we have clarified this point and have enhanced our discussion on negative enrichment of the STARR-seq output coverage in the revised manuscript (Excerpt 2.7.4). In short, STARRPeaker is not designed for detecting negative enrichment. |

REF2.7.11 – (Minor): NB2

| Referee Comment | Minor comments:  11. Using NB2 to refer to the negative binomial is not explained, and it cannot be assumed that most readers are aware that there are two forms of the NB distribution. This should be explained and cited, or replaced with a simple "NB". |
| --- | --- |
| Author Response | We thank the referee for this comment. We have revised the manuscript to explain the rationale behind the use of NB2 (Excerpt 2.7.11). In summary, the NB2 notion was first introduced by Cameron and Trivedi [15], and the difference between NB1 and NB2 lies in the conditional variance of 𝑦_%_ . The most common implementation of the negative binomial is, counterintuitively, NB2; therefore, we used NB2 instead of NB1. |
| Excerpt 2.7.11 (main text) | “… |

|  | A negative binomial is a generalization of a Poisson regression that allows the variance to be different from the mean, shaped by the dispersion parameter 𝜃. There are two alternative forms of parametrization for a negative binomial – NB1 and NB2 – which were first introduced by Cameron and Trivedi [36]. The difference between NB1 and NB2 is in the conditional variance of 𝑦_%_. Assuming 𝑦_%_ has mean 𝜆_%_, the general variance function follows the form 𝜔_%_ = 𝜆_%_ + 𝛼𝜆_%_^T^, where 𝛼 is a scalar parameter. NB1 uses 𝑝 = 1, whereas NB2 uses the quadratic form of variance with 𝑝 = 2. We use the most common implementation of the negative binomial, NB2, hereafter.  …” |
| --- | --- |

REF2.7.12 – (Minor): TF ChIP-seq accession

| Referee Comment | Minor comments:  12. Table S3 is missing accessions for the TF ChIP-seq used to generate Figure 5D |
| --- | --- |
| Author Response | In the revised manuscript, we added a list of used TF ChIP-seq accessions to Supplementary Table S3. |

REF2.7.13 – (Minor): alternate mode

| Referee Comment | Minor comments:  13. The authors describe an alternative mode of operation of their method, "mode 2", but do not explain when this is useful for users to choose |
| --- | --- |
| Author Response | We have added more discussion regarding this point in the revised manuscript (Excerpt 2.7.13). |
| Excerpt 2.7.13 (main text) | “… |
|  | Alternatively, instead of using the input library variable 𝑡_%_ as one covariate, we can directly use it as an offset variable. Generally, a |
|  | fractional observation cannot be modeled using discrete probability. However, an offset variable in a generalized linear model can be |
|  | used to correct the response term to behave like a fraction. One advantage of using the input variable as an “exposure” to the RNA |
|  | output coverage is that it allows us to directly model the basal transcription rate (the ratio of RNA to DNA) as a rate response variable. |
|  | More details on this alternative parametrization are included in the Methods section. |
|  | … |
|  | The natural log of 𝑡_%_ on the RHS ensures 𝜇_%_ is normalized in the model, acting as an offset variable. In STARRPeaker software, we |
|  | allow users to optionally choose this alternative rate model (implemented as “mode 2”) instead of the default covariate model described |
|  | in the main text. This alternate model is useful if constant basal transcription is expected throughout the genome or if covariates are |
|  | available for directly modelling the basal transcription rate 𝜋. |
|  | …” |

REF2.7.14 – (Minor): mode 2

| Referee Comment | Minor comments:  14. Related to that, it is somewhat confusing to see a fractional observation modeled with a negative binomial (line 432). Please extend the discussion to explain this. |
| --- | --- |
| Author Response | We have clarified this point in the revised manuscript (Excerpt 2.7.13). |

References

1. Pennacchio LA, Bickmore W, Dean A, Nobrega MA, Bejerano G. Enhancers: five essential questions. Nat Rev Genet [Internet]. NIH Public Access; 2013 [cited 2019 Sep 28];14:288–95. Available from: <http://www.ncbi.nlm.nih.gov/pubmed/23503198>
2. Muerdter F, Boryń ŁM, Woodfin AR, Neumayr C, Rath M, Zabidi MA, et al. Resolving systematic errors in widely used enhancer activity assays in human cells. Nat Methods [Internet]. Nature Publishing Group; 2018 [cited 2019 Sep 11];15:141–9. Available from: <http://www.nature.com/articles/nmeth.4534>
3. Liu Y, Yu S, Dhiman VK, Brunetti T, Eckart H, White KP. Functional assessment of human enhancer activities using whole-genome STARR-sequencing. Genome Biol [Internet]. 2017 [cited 2019 Jul 5];18:219. Available from: https://genomebiology.biomedcentral.com/articles/10.1186/s13059-017-1345-5
4. Wang X, He L, Goggin SM, Saadat A, Wang L, Sinnott-Armstrong N, et al. High-resolution genome-wide functional dissection of transcriptional regulatory regions and nucleotides in human. Nat Commun. Nature Publishing Group; 2018;9:1–15.
5. Benjamini Y, Hochberg Y. Controlling the false discovery rate: a practical and powerful approach to multiple testing. J R Stat Soc Ser B [Internet]. John Wiley & Sons, Ltd (10.1111); 1995 [cited 2019 Jul 5];57:289–300. Available from: <http://doi.wiley.com/10.1111/j.2517-> 6161.1995.tb02031.x
6. Huang L, Zhang H, Deng D, Zhao K, Liu K, Hendrix DA, et al. LinearFold: linear-time approximate RNA folding by 5’-to-3’ dynamic programming and beam search. Bioinformatics [Internet]. Narnia; 2019 [cited 2019 Sep 11];35:i295–304. Available from: https://academic.oup.com/bioinformatics/article/35/14/i295/5529205
7. Lorenz R, Bernhart SH, Höner zu Siederdissen C, Tafer H, Flamm C, Stadler PF, et al. ViennaRNA Package 2.0. Algorithms Mol Biol [Internet]. BioMed Central; 2011 [cited 2020 Mar 13];6:26. Available from: https://almob.biomedcentral.com/articles/10.1186/1748-7188-6-26
8. Mathews DH, Disney MD, Childs JL, Schroeder SJ, Zuker M, Turner DH. Incorporating chemical modification constraints into a dynamic programming algorithm for prediction of RNA secondary structure. Proc Natl Acad Sci U S A. National Academy of Sciences; 2004;101:7287–92.
9. Papoulis A, Athanasios. Probability, random variables and stochastic processes. New York McGraw-Hill, 1984, 2nd ed [Internet]. 1984 [cited 2019 Jul 5]; Available from: [http://adsabs.harvard.edu/abs/1984prvs.book.](http://adsabs.harvard.edu/abs/1984prvs.book)	P
10. Pang B, Snyder MP. Systematic identification of silencers in human cells. Nat Genet [Internet]. Nature Publishing Group; 2020 [cited 2020 Mar 13];52:1–10. Available from: <http://www.nature.com/articles/s41588-020-0578-5>
11. Kawaji H, Kasukawa T, Forrest A, Carninci P. The FANTOM 5 collection, a data series underpinning mammalian transcriptome atlases in diverse cell types. Sci Data. 2017;2016–8.
12. Inoue F, Kircher M, Martin B, Cooper GM, Witten DM, McManus MT, et al. A systematic comparison reveals substantial differences in chromosomal versus episomal encoding of enhancer activity. Genome Res [Internet]. 2017 [cited 2019 Jul 6];27:38–52. Available from: <http://www.ncbi.nlm.nih.gov/pubmed/27831498>
13. Klein JC, Agarwal V, Inoue F, Keith A, Martin B, Kircher M, et al. A systematic evaluation of the design, orientation, and sequence context dependencies of massively parallel reporter assays. bioRxiv [Internet]. Cold Spring Harbor Laboratory; 2019 [cited 2019 Mar 15];576405.

Available from: https:[//www.biorxiv.org/content/10.1101/576405v1](http://www.biorxiv.org/content/10.1101/576405v1)

14. Rathert P, Roth M, Neumann T, Muerdter F, Roe J-S, Muhar M, et al. Transcriptional plasticity promotes primary and acquired resistance to BET inhibition. Nature [Internet]. 2015 [cited 2019 Jul 5];525:543–7. Available from: <http://www.ncbi.nlm.nih.gov/pubmed/26367798>
15. Cameron ACA, Trivedi PK. Regression Analysis of Count Data [Internet]. Cambridge: Cambridge University Press; 2013 [cited 2019 Jul 5]. Available from: <http://ebooks.cambridge.org/ref/id/CBO9781139013567>

**Second round of review**

**Reviewer 1**

The manuscript has been gretly improved and all my remarks have been satisfactorily adressed

**Reviewer 2**

The authors have addressed several of the concerns raised in the previous round. We do however find several concerns (remaining, or as a result from the revision), listed below.


--------------------------
Major Comments:
--------------------------

Datasets:
1.     Additional analyses using external datasets were included in the revised manuscript. However, these new datasets are only included in a single section, and the sections dedicated to the properties of STARR-seq data, model evaluation and comparing with other methods are still relying solely on this study’s data. To show the generalizability of the method and its assumptions, please add the missing analyzes for the additional datasets. Specifically:
    a.     It should be demonstrated that the properties of STARR-seq data described in figures 2 and 3 are consistent across different STARR-seq protocols and datasets.
    b.     It should be demonstrated that the conclusions regarding the performance of STARRPeaker compared with alternative methods holds for external datasets and protocols as well.


Model Evaluation
1.     We are missing an analysis of how changing the parameters affects the results, as well as guidance for users on how to choose these parameters (since STARR-seq experiments do not necessarily share the same design). The response to a similar criticism on the previous review states that these parameters were determined based on experimental design – but no further details are provided.
2.     The method assumes that most regions will have basal transcription rate (L273). However, we are missing a demonstration that the model is in fact well-calibrated under this assumption. For instance, the authors should show that the p-value distribution matches the theoretical behavior (most values are uniformly distributed, with an enrichment of low values for cases where the null is rejected).
3.     In the initial evaluation (L335) the authors observe high correlation between replicates, but the meaning of this correlation is not explained. Is this correlation at the level of raw data? or between the results of applying STARRPeaker to the two replicates? What is the meaning of the axes of the figure?
4.     Subsequently, L339 states the number of peaks in the overlap between replicates, but does not specify what fraction of peaks overlap. To demonstrate the robustness of the method, the authors should specify how many peaks were called in total, and how many of those were called in both replicates.
5.     Supplementary Figure 4 demonstrates that the method is robust to suboptimal datasets, which is an important analysis. However, several details are missing from it:
    a.     Which dataset was used for this analysis?
    b.     How the was data subsampled? (e.g., Before or after alignment of the reads?)
    c.     Which libraries were subsampled? Only input, only output, or both? (note that it would be worth exploring multiple scenarios, since sequencing depth is not identical in many studies, and understanding if STARRPeaker is sensitive to data quality could be important, especially in genome-wide assays).
    d.     The figure shows that a total of 5000 peaks were used for this analysis, but the authors report more peaks than that in previous sections. Please explain this discrepancy.


Orientation Bias:
1.     The analysis concludes that there is insufficient evidence for bias. However, the results show that thousands of enhancers have significant strand bias, more than the expected false discovery rate. Could this not be used as sufficient evidence that this bias does exist?
2.     Please evaluate the extent of bias with additional datasets to show that this conclusion holds.
3.     If strand bias can be incorporated into the STARRPeaker model, it would be of interest to test if this covariate does not improve model performance (using a similar approach to supplementary figure 2).
4.     Some statements in this analysis could use clarification:
    a.     L373: “less than one third of the enhancers (n=690)…” – one third of which group? The numbers mentioned previously discuss, for example, 1,605 enhancers representing 3.19% of the total.
    b.     Please provide a complete description of the test
    c.     What is the meaning of the axes in Supp. Figure 5?


Comparison to other methods:
1.     The simulation procedure should be explained in full (currently there is only a brief mentioning of it the main text and no methods section, which leaves many unknowns).
2.     Please extend the simulation to more than a handful of peaks. The simulated dataset should be demonstrated to have similar statistical properties as real STARR-seq data and be used to evaluate both sensitivity and specificity.
3.     “We used a uniform P-value threshold of 0.001 and subsampled the peaks before the comparison”
    a.     The rationale for subsampling peaks is not clear. The tradeoff between sensitivity and specificity, and how each method chooses to position itself in this regard, is a major difference between the methods that should be part of the comparison. Comparing all of the results from one method to a fraction of the results from another method (which seems to be the case here) may not be fair
    b.     Please report the overlap between the methods in addition to the number of peaks each method called (Supp. Table S4).
    c.     While we accept the rationale for using the default parameters for basicSTARRseq, using the default parameters for MACS2 is harder to justify as it was not designed for STARR-seq. This is an important point since MACS2 documentation explicitly provides different sets of optimal parameters for different types of data, since peak characteristics are different.


External datasets
1.     The definition of “percent overlap” is confusing. Is this the percent of peaks called that exist in the validation set, or the percent of the peaks in the validation set that were called?
2.     It would be useful for the authors to report both numbers, and even construct actual ROC/PR curves using the validation set as the ground truth. This would also make the non-standard p-value threshold (0.001) and subsampling of results unnecessary.
3.     Please provide a more complete description of the. results in Figure S8: STARRPeaker correctly calls 6 of the 9 peaks, but also calls several other regions that are not peaks. The false-positive rate seems to be much higher in the competing methods, but the authors don’t mention this either.
4.     Application to external datasets
    a.     Figure S9B seems to suggest that the results attained from basicSTARRSeq and some post-hoc filtering are superior to the STARRPeaker results, using the same enrichment metric used throughout the manuscript. This could be due to peaks that STARRPeaker missed that would increase the enrichment, or additional peaks that STARRPeaker called that are decreasing the enrichment. Please comment on this.
    b.     The authors apply STARRPeaker to a Cap-STARR-seq dataset, and report that STARRPeaker calls less than 25% of the published peaks, but provide no further exploration regarding which set of results is superior. It is then concluded that STARRPeaker can be “reliably applied” but the meaning of this term is not explained.
    c.     When applying STARPeaker to a third dataset, the authors report that the method recovers a smaller set of peaks, concluding that STARRPeaker is highly specific, and more robust and reliable. However, as stated earlier in the manuscript, high specificity is often traded for low sensitivity, and it is not clear that this isn’t the case here. In addition to the peaks STARRPeaker calls, there are 149 additional peaks that are called by both competing methods but not by STARRPeaker. The authors should justify the claim that these are false positives of the other methods and not false negatives of STARRPeaker.


--------------------------
Minor Comments:
--------------------------
1.     Figure 1:
    a.     “We found that the position of the peaks shifted up approximately 200 bp when we used the alternate model (Figure 1A, Supplementary Figure 1A). Such a shift caused by the read-based coverage could lead to the omission of TF binding sites located at the boundary. “
    I am assuming that the label “r1” in figure 1A indicates the beginning of read 1. In that case, isn’t it expected – by definition – to see a shift in the peak centers?
    b.     “peaks calculated based on the alternate model had lower fold enrichment and were less confident “
    The terms fold enrichment and the procedure for estimating confidence have not been defined up to this point of the manuscript.
    c.     Generally, we find that the comparison to a “read- ends” quantification lacks in rigor. However, we do see this as a minor point. We suggest that the authors remove the less substantiated statements (e.g., “The benefit of using the center of the fragment is highlighted in Figure 1E, where we find more concise and precise peak”) and shorten this section.

2.     Figure 2: which dataset is being used there?
3.     Supplementary Figure 2: the QQ-plots in panel A are not explained or discussed. How were the theoretical quantiles computed?
4.     Supplementary Figure 2: please define the covariate abbreviations.
5.     Figures 2 and 3 seem to use a single dataset, but it is not clear which one (HepG2 or K562) and if both replicates were used or only a single one.
6.     Line387: “shifted toward the enrichment of sequencing reads ”. Not clear what this mean.
7.     The authors replaced most instances of “STARR-seq coverage” with “output library coverage”, but this term remains in the figure labels of figures 2-4.
8.     Figure 5: what is the control in the histone modification enrichment?
9.     Figure 6: which numerator was used for the % overlap (20k or the number of peaks in the published/ reference dataset)?
10.     When discussing the comparative advantages of STARR-seq to MPRA, please mention the trade-off between the methods: STARR-seq can be made genome-wide and unbiased, but is limited to sequences that already exist in the genome, whereas MPRA is more limited in scale but enable testing of perturbed/mutated sequences, or multiple alleles in the same system.
11.     It is stated that “the adjusted p-value should be regarded as the likelihood of a candidate region being an enhancer” (L247). This interpretation of p-values is misleading.  p-values are likelihood under the null hypothesis, not under the alternative, so they would be inversely related to the likelihood of a candidate region being an enhancer.
12.     (L307) The term \hat{beta} seems to be a typo.
13.     In Supplemental Figure 3, the authors show a high correlation between replicates, but it is not clear what exactly is correlated. Is this correlation of effect size after peak-calling using STARRPeaker, or correlation of the data itself? How was this computed?
14.     An alternative mode is discussed in L285. While this explanation was revised, it is still unclear what the benefit of this alternative mode, when should users consider using it, and how it compares with the default mode. The authors should either expand on this point or avoid mentioning this alternative entirely.

**Authors’ response to reviewers**

### REF1.0 – Overall comments

| Referee  Comment | Reviewer #1: The manuscript has been greatly improved and all my remarks have been satisfactorily addressed |
| --- | --- |
| Author  Response | We thank the referee for the positive comment. |

### REF2.0 – Overall comments

| Referee  Comment | Reviewer #2: The authors have addressed several of the concerns raised in the previous round. We do however find several concerns (remaining, or as a result from the revision), listed below. |
| --- | --- |
| Author  Response | We thank the referee for the thoroughly reviewing this study and providing constructive feedback. We carefully went through all of the comments. In the revised manuscript, we expanded our analysis and clarified all points raised in this report. The following list summarizes the new and updated figures and tables in this revision:   - (updated) Figure 1 - (updated) Figure 2 - (updated) Figure 3 - (updated) Figure 4 - (updated) Supplementary Figure S1 - (new) Supplementary Figure S2 Extension of Figure 2 - (new) Supplementary Figure S4 Extension of Figure 3 - (new) Supplementary Figure S5 Q-Q plot of P-values - (updated) Supplementary Figure S7 - (updated) Supplementary Figure S8 - (updated) Supplementary Figure S9 - (updated) Supplementary Figure S11 - (updated) Supplementary Figure S12 - (updated) Supplementary Table S4 - (new) Supplementary Table S5 Sensitivity analysis of using alternative parameters - (new) Supplementary Table S6 List of control regions in the MYC locus for simulation - (new) Supplementary Table S7 Sensitivity and specificity analysis using simulated STARR-seq data - (new) Supplementary Table S8 Sensitivity and specificity analysis using the Fulco et al. 2016 CRISPRi dataset |

### REF2.1.1 – Datasets A

| Referee  Comment | 1. Additional analyses using external datasets were included in the revised manuscript. However, these new datasets are only included in a single section, and the sections dedicated to the properties of STARR-seq data, model evaluation and comparing with other methods are still relying solely on this study’s data. To show the generalizability of the method and its assumptions, please add the missing analyzes for the additional datasets. Specifically:  a. It should be demonstrated that the properties of STARR-seq data described in figures 2 and 3 are consistent across different STARR-seq protocols and datasets. |
| --- | --- |
| Author  Response | We thank the referee for these thoughtful suggestions. Accordingly, we have expanded the analyses on the methodology and the underlying assumptions using the additional datasets. We added two new Supplementary Figures shadowing the analyses described in Figures 2 and 3. Based on additional analyses on previously published STARR-seq datasets, we confirmed that the properties of the STARR-seq data described in both Figure 2 and 3, especially the covariates are consistent across different STARR-seq protocols and datasets (Excerpt 2.1.1-3). |
| Excerpt 2.1.1 (main text) | “…  We next tested whether potential sequencing biases and other covariates confounded STARR-seq readouts (Figure 2 and Supplementary Figure S2).  …  To demonstrate the generalizability of the method and assumptions, we tested the model fit against previously published datasets that utilized different STARR-seq protocols (Supplementary Figure S4). We observed consistent properties of the STARR-seq data across the different STARR-seq protocols and datasets.  …” |
| Excerpt 2.1.2 (suppl. fig. S2) |  |
| Excerpt 2.1.3 (suppl. fig. S4) |  |

### REF2.1.2 – Datasets B

| Referee  Comment | b. It should be demonstrated that the conclusions regarding the performance of STARRPeaker compared with alternative methods holds for external datasets and protocols as well. |
| --- | --- |
| Author  Response | As suggested by the referee, we have strengthened the section titled “Application to external STARR-seq datasets” where we compared the performance of STARRPeaker to alternate methods using external datasets through a series of analyses (Excerpt 2.1.2). We demonstrated that STARRPeaker consistently finds less false positives and more robust and reproducible peaks in terms of finding more common regulatory elements across multiple external datasets. Additionally, we showed that our method does not require additional filtering or manual curation, unlike many other methods. We acknowledge that the true performance (accuracy) of a method can only be evaluated from the ground truth (controls), yet there are no gold standards established for enhancers in general. However, we think that a method should also be evaluated qualitatively based on how accurately it models the underlying data and how reliable the test statistics are. |
| Excerpt 2.1.2 (main text) | “…  *Application to external STARR-seq datasets*  To ensure that STARRPeaker can be generally applied to different variants of STARR-seq assays, we tested STARRPeaker on previously published STARR-seq datasets.  …” |

### REF2.2.1 – Model Evaluation A

| Referee  Comment | 1. We are missing an analysis of how changing the parameters affects the results, as well as guidance for users on how to choose these parameters (since STARR-seq experiments do not necessarily share the same design). The response to a similar criticism on the previous review states that these parameters were determined based on experimental design – but no further details are provided. |
| --- | --- |
| Author  Response | As suggested by the referee, we added a sensitivity analysis showing the effects of adjusting the parameters on the results (Excerpt 2.2.1). We provided more details and rationale for our choice of parameters as well as guidance on how to choose parameters for other STARR-seq experiments in the Methods section. |
| Excerpt 2.2.1 (main text) | “…  **Recommended parameters for the model**  We determined the model parameters from the experimental design of the STARR-seq assay. Our STARR-seq input library was based on DNA fragments that were size-selected on an agarose gel for 500 bp. Therefore, we defined the bin size to be 500 bp with a step size of 100 bp. Furthermore, we chose the minimum and maximum template size to be 200 and 1,000 bp, respectively, to recover RNA generated from the STARR-seq input library. We recommend setting the minimum template size no less than 200 bp, as a shorter template can become more prone to PCR bias during sequencing. Based on the sequencing depth, we determined the minimum coverage for the whole genome to be 10. To guide users on how to choose these parameters, we provide a sensitivity analysis of how changing the parameters affects the results (Supplementary Table S5). Overall, more than 80% of peaks were consistent regardless of varying the parameters. We found that changing the bin size affected the average peak size and changing the step size affected the resolution. However, we found that using a 50 bp step size approximately doubled the processing time.  …” |

### REF2.2.2 – Model Evaluation B

| Referee  Comment | 2. The method assumes that most regions will have basal transcription rate (L273). However, we are missing a demonstration that the model is in fact well-calibrated under this assumption. For instance, the authors should show that the p-value distribution matches the theoretical behavior (most values are uniformly distributed, with an enrichment of low values for cases where the null is rejected). |
| --- | --- |
| Author  Response | As suggested, we added new Q-Q plots of the P-value distribution to show that most P-values are uniformly distributed in Supplementary Figure S5 (Excerpt 2.2.2.1 and 2.2.2.2). In addition, we evaluated how well the model is calibrated in Supplementary Figure S3. We demonstrated through a Q-Q plot of residuals that the sample quantile matches the theoretical quantile. |
| Excerpt 2.2.2.1 (main text) | “…  *Initial evaluation of STARRPeaker enhancers*  Our model assumes that most genomic regions have a basal level of transcription activity. To validate that our model is well calibrated under this assumption, we examined the P-value distribution against a theoretical uniform distribution. As expected, most observed P-values followed the null distribution, and the P-values only deviated from expectation for very low values (Supplementary Figure S5).  …” |
| Excerpt 2.2.2.2 (suppl. fig. S5) |  |

### REF2.2.3 – Model Evaluation C

| Referee  Comment | 3. In the initial evaluation (L335) the authors observe high correlation between replicates, but the meaning of this correlation is not explained. Is this correlation at the level of raw data? or between the results of applying STARRPeaker to the two replicates? What is the meaning of the axes of the figure? |
| --- | --- |
| Author  Response | We thank the referee for pointing this out. We clarified the meaning of correlation in the revised manuscript. We calculated the correlation using the intermediate fragment count data. The X- and Y-axis represent the natural log of the fragment counts. We added a detailed description of how we calculated the Pearson correlation in the revised manuscript (Excerpt 2.2.3) |
| Excerpt 2.2.3 (main text) | “…  **Supplementary Figure S6** Correlation of RNA fragment coverage between replicates for **(A)** HepG2 or **(B)** K562 cell lines. Pairwise coverage was calculated using 1,000 bp bins across the genome. The X- and Y-axis represent the natural log of fragment counts. A pseudo count of 1 was added to the fragment counts for plotting only. Bins with abnormally high fragment counts were removed to avoid inflation of the Pearson correlation. We used the median absolute deviation method with a scaling factor of 200 to filter extremely large deviations from the median.  …” |

### REF2.2.4 – Model Evaluation D

| Referee  Comment | 4. Subsequently, L339 states the number of peaks in the overlap between replicates, but does not specify what fraction of peaks overlap. To demonstrate the robustness of the method, the authors should specify how many peaks were called in total, and how many of those were called in both replicates. |
| --- | --- |
| Author  Response | As suggested by the referee, we specified how many peaks were called in total and how many of those were called in both replicates (Excerpt 2.2.4). |
| Excerpt 2.2.4 (main text) | “…  We processed two biological replicates from each cell type independently and assessed the correlation between each pair (see Methods for detail). We identified 50,389 and 52,927 candidate enhancers from HepG2 replicate 1 and 2, and by consolidating peaks from both replicates, we identified 32,929 (65.3% of rep 1; 62.2% of rep2) reproducible candidate enhancers from the HepG2 cell line. Similarly, by consolidating 30,194 and 41,810 candidate enhancers from K562 replicate 1 and 2, we identified 20,471 (67.8% of rep 1; 49.0% of rep 2) reproducible candidate enhancers from the K562 cell line (Supplementary Table S1). Overall, we observed high correlation of RNA fragment coverage between two replicates (PCC=0.99 for both HepG2 and K562; see Supplementary Figure S6). This result indicates the correlation is dominated by negatives.  …” |

### REF2.2.5.1 – Model Evaluation E

| Referee  Comment | 5. Supplementary Figure 4 demonstrates that the method is robust to suboptimal datasets, which is an important analysis. However, several details are missing from it:  a. Which dataset was used for this analysis? |
| --- | --- |
| Author  Response | We used HepG2 WG-STARR-seq for this analysis. We have updated the section titled “Assessment of robustness and reproducibility of the method” with more details (Excerpt 2.2.5.1). Please note that former Supplementary Figure 4 has been relabeled as S7. |
| Excerpt 2.2.5.1 (main text) | “…  *Assessment of robustness and reproducibility of the method*  A reliable peak-calling method allows one to identify stable peaks from suboptimal datasets. To evaluate the robustness of the STARRPeaker method, we generated random subsets of HepG2 whole-genome STARR-seq library after aligning the reads and compared the quality of the peak calls. We subsampled randomly at various rates from 20 to 80% of the total dataset. We found that STARRPeaker was able to reliably identify approximately 90% of the candidate enhancers (consolidated) using 80% of the original sequencing library and 80% of the candidate enhancers using 40% of the original sequencing library (Supplementary Figure S7A). When we focused on strong enhancer candidates, approximately 98% of the top 5,000 enhancers were recovered using only 60% of the original sequencing library (Supplementary Figure S7B). However, the quality of the peak calls started to deteriorate when 40% or less were used.  …” |

### REF2.2.5.2 – Model Evaluation F

| Referee  Comment | b. How the was data subsampled? (e.g., Before or after alignment of the reads?) |
| --- | --- |
| Author  Response | We generated random subsets of the HepG2 whole-genome STARR-seq library after aligning the reads (Excerpt 2.2.5.1). |

### REF2.2.5.3 – Model Evaluation G

| Referee  Comment | c. Which libraries were subsampled? Only input, only output, or both? (note that it would be worth exploring multiple scenarios, since sequencing depth is not identical in many studies, and understanding if STARRPeaker is sensitive to data quality could be important, especially in genome-wide assays). |
| --- | --- |
| Author  Response | We only generated random subsamples of the output library. We believe fixing the input library constant was a more fair way to evaluate the true effect of subsampling. |

### REF2.2.5.4 – Model Evaluation H

| Referee  Comment | d. The figure shows that a total of 5000 peaks were used for this analysis, but the authors report more peaks than that in previous sections. Please explain this discrepancy. |
| --- | --- |
| Author  Response | As suggested, we expanded the analysis and added a new supplementary figure (Excerpt 2.2.5.4) that shows the effect of subsampling using all of the consolidated candidate enhancers. As the referee pointed out, we originally focused on only a subset of 5,000 enhancers to illustrate our point. In our new analysis, consistent with our previous findings, STARRPeaker was able to reliably identify approximately 90% of the candidate enhancers (consolidated) using 80% of the original sequencing library and 80% of candidate enhancers using 40% of the original sequencing library (Excerpt 2.2.5.1). |
| Excerpt 2.2.5.4 (suppl. fig. S7) |  |

### REF2.3.1 – Orientation Bias A

| Referee  Comment | 1. The analysis concludes that there is insufficient evidence for bias. However, the results show that thousands of enhancers have significant strand bias, more than the expected false discovery rate. Could this not be used as sufficient evidence that this bias does exist? |
| --- | --- |
| Author  Response | We thank the referee for this comment. We carefully reviewed this issue and strengthened the orientation bias analysis. The original analysis was performed for each replicate and only one third of those biases were consistent across replicates. What we overlooked in the original analysis was that the input genomic DNA fragments could also have strand biases. Having a skewed representation of DNA, the resulting RNA might be due to PCR artifacts rather than orientation-specific activities. Considering this possibility, we revised our method and re-evaluated potential strand biases among the candidate enhancers we identified (Excerpt 2.3.1). Our new results suggest that the majority of the candidate enhancers (>95%) do not show orientation-specific activities, consistent with our previous findings. |
| Excerpt 2.3.1 (main text) | “…  *Evaluation of potential orientation bias in candidate enhancers*  In general, enhancers are thought to function independent of orientation [40]. However, the fragment counts in one orientation could be skewed over the other due to orientation-specific activities, PCR, or sequencing artifacts. To test for potential orientation-based biases, we ran a series of binomial tests on the candidate enhancers we identified and evaluated for possible orientation-specific activities (see Methods). We observed a small fraction of candidate enhancers showing orientation bias [2.58% for HepG2 (n=850); 4.49% for K562 (n=919); FDR ≤ 0.01] in both replicates (Supplementary Figure S8). Furthermore, only a few candidates showed extreme bias [n=3 for HepG2; n=4 for K562; >90% fragments on one strand]. Thus, true orientation-dependent activities are unlikely in our STARR-seq data, but that the orientation may have an effect on the efficiency. These findings provide further support that enhancers function independent of orientation.  …” |

### REF2.3.2 – Orientation Bias B

| Referee  Comment | 2. Please evaluate the extent of bias with additional datasets to show that this conclusion holds. |
| --- | --- |
| Author  Response | As suggested by the referee, we re-evaluated the extent of bias and strengthened our analysis in the revised manuscript. We provide more details on the new analysis in the Methods section (Excerpt 2.3.2). |
| Excerpt 2.3.2 (main text) | “…  **Evaluation of potential orientation bias**  For all enhancer peaks identified from both the HepG2 and K562 cell lines, we evaluated if there was an overrepresentation of fragments in a specific orientation. If there is no orientation bias, the STARR-seq active region should be equally represented by both forward- and reverse-stranded fragments. We performed a binomial test for the statistical significance on how unlikely it is to have a fragment distribution skewed on one strand.  $\left( \begin{matrix} n \\ k \end{matrix} \right)p^{k}\left( 1-p \right)^{n-k}$  where $p$ is 0.5 (equal chance of being either forward or reverse stranded), $n$ is number of all supporting fragments, and $k$ is the number of forward-stranded fragments. We adjusted P-values using FDR (BH) and used 0.01 as a cutoff. We ran binomial tests for both replicates. To call a region orientation-biased, we ensured that genomic DNA fragments were equally represented in both forward and reverse strands, and we checked for significant strand bias in both replicates. If strand bias is already present in a genomic DNA library, it is more likely that the bias will be due to amplification by PCR rather than being a result of orientation-specific activity.  …” |

### REF2.3.3 – Orientation Bias C

| Referee  Comment | 3. If strand bias can be incorporated into the STARRPeaker model, it would be of interest to test if this covariate does not improve model performance (using a similar approach to supplementary figure 2). |
| --- | --- |
| Author  Response | We thank the referee for this suggestion. We considered this possibility and we agree that bias could potentially be used as a covariate to improve model performance. However, we did not find enough evidence that strand bias affects STARR-seq experiments. Moreover, we found it difficult to use strand bias as a covariate because the bias does not have directionality (i.e., one orientation is not preferred over the other across the assay) and neither strandedness nor the extent of bias correlated with STARR-seq readouts. Please note that former Supplementary Figure 2 has been relabeled as S3. |

### REF2.3.4.1 – Orientation Bias D

| Referee  Comment | 4. Some statements in this analysis could use clarification:  a. L373: “less than one third of the enhancers (n=690)…” – one third of which group? The numbers mentioned previously discuss, for example, 1,605 enhancers representing 3.19% of the total. |
| --- | --- |
| Author  Response | We thank the referee for these suggestions. To answer the referee’s question, previously, we referred to the group of enhancers with potential orientation-specific activities. However, we have re-written the section to include the new analysis in the revised manuscript (Excerpt 2.3.1). |

### REF2.3.4.2 – Orientation Bias E

| Referee  Comment | b. Please provide a complete description of the test |
| --- | --- |
| Author  Response | As suggested by the referee, we added more details about the test to the Methods section (Excerpt 2.3.2). |

### REF2.3.4.3 – Orientation Bias F

| Referee  Comment | c. What is the meaning of the axes in Supp. Figure 5? |
| --- | --- |
| Author  Response | We have re-plotted Supplementary Figure S8 (Excerpt 2.3.4). Each dot represents a peak and the axes show fragment counts attributed from either forward- or reverse-stranded fragments. Please note that former Supplementary Figure 5 has been relabeled as S8. |
| Excerpt 2.3.4 (supp. fig. S8) |  |

### REF2.4.1 – Comparison to other methods A

| Referee  Comment | 1. The simulation procedure should be explained in full (currently there is only a brief mentioning of it the main text and no methods section, which leaves many unknowns). |
| --- | --- |
| Author  Response | As suggested by the referee, we added a new section detailing how we generated and performed the simulation to the Methods section (Excerpt 2.4.1). |
| Excerpt 2.4.1 (main text) | “…  **Simulation of STARR-seq dataset**  We created an artificial STARR-seq dataset where the ground truth exists. We used the original STARR-seq input library as the base of the simulation. We focused on an approximately 2.6 Mbp region spanning the MYC locus (chr8:127128459-129731914). We artificially selected 28 control regions equally sized at 500 bp. Among these control regions, we included coordinates of four *in vivo* MYC enhancers identified from Fulco et al., 2016 (MYC-e1, chr8:127898897-127898963; MYC-e2, chr8:127960392-127960502; MYC-e5, chr8:129581781-129582461; MYC-e6, chr8:129689361-129689694) [1]. We selectively generated paired-end reads that only support control regions. Since we expect the background regions to have a basal level of transcription, we matched the read distribution of the input library to make the transcription rate equal to 1. All 28 control regions were then merged to create an artificial STARR-seq output library, and finally, the read coverage was visually inspected to ensure that it resembles one from the actual STARR-seq dataset.  …” |

### REF2.4.2 – Comparison to other methods B

| Referee  Comment | 2. Please extend the simulation to more than a handful of peaks. The simulated dataset should be demonstrated to have similar statistical properties as real STARR-seq data and be used to evaluate both sensitivity and specificity. |
| --- | --- |
| Author  Response | As suggested by the referee, we have expanded the simulation in the revised manuscript. We created a simulated STARR-seq dataset that includes 28 control regions (the ground truth) and used this to evaluate both sensitivity and specificity of three peak-calling methods (Excerpt 2.4.2.1 and 2.4.2.2). Please note that former Supplementary Figure 6 has been relabeled as S9. |
| Excerpt 2.4.2.1 (main text) | “…  First, we qualitatively assessed the peak-calling algorithms using a simulated dataset where the ground truth exists. The simulation was primarily designed for positives in the dataset, and as a result, it emphasizes the sensitivity (as opposed to specificity) of each method. We created an artificial STARR-seq dataset that contains 28 spike-in controls; a hybrid of DNA input and RNA output libraries with active elements at pre-defined loci (Supplementary Table S6; see Methods for details). All three methods successfully identified peaks at 28 control regions (Supplementary Figure S9). However, we noticed that BasicSTARRseq peaks were fragmented and off-centered from the controls due to its limitations of fixed peak size and read-based coverage calculation. As a result, several false-positive peaks were called adjacent to the controls. We calculated the sensitivity and specificity of each method using 28 controls as the gold standard (Supplementary Table S7). We considered a result positive if at least 80% of the peak overlapped with the control, and we assumed the presence of approximately 1,000 true negative regions. Both STARRPeaker and MACS2 had 100% sensitivity and specificity, whereas BasicSTARRseq had 75% sensitivity and 94.9% specificity.  …” |
| Excerpt 2.4.2.2 (suppl. fig. S9) |  |

### REF2.4.3.1 – Comparison to other methods C

| Referee  Comment | 3. “We used a uniform P-value threshold of 0.001 and subsampled the peaks before the comparison”  a. The rationale for subsampling peaks is not clear. The tradeoff between sensitivity and specificity, and how each method chooses to position itself in this regard, is a major difference between the methods that should be part of the comparison. Comparing all of the results from one method to a fraction of the results from another method (which seems to be the case here) may not be fair |
| --- | --- |
| Author  Response | We think there is a bit of confusion here, so we tried to explain our rationale better in the revised manuscript (Excerpt 2.4.3.1). We are not comparing all results from one method to a fraction of results from another method. In a simple overlap calculation, there is a clear advantage for the method that has more peaks. For example, suppose each method generates a set of randomized peaks. If one method has 20 times more peaks than the other, it is likely to have more overlap solely by chance. To eliminate this artifact, we subsampled peaks down to the same number and compared the overlap with external datasets. |
| Excerpt 2.4.3.1 (main text) | “…  While it is uncertain how many true enhancers were present in each sample, we had to ensure that we make a fair comparison across different methods due to the tradeoff between sensitivity and specificity. An increase in sensitivity is generally achieved at the expense of a decrease in specificity, as described in receiver operating characteristic curves. In our context, a method having higher specificity suffers from having less overlap with open chromatin and previously identified enhancers from other assays. Suppose each method is generating a set of randomized peaks, then the method with the greater number of peaks is likely to have more overlap solely by chance. To eliminate this artifact, we used a uniform P-value threshold of 0.001 and subsampled the peaks down to the same number before the comparison.  …” |

### REF2.4.3.2 – Comparison to other methods D

| Referee  Comment | b. Please report the overlap between the methods in addition to the number of peaks each method called (Supp. Table S4). |
| --- | --- |
| Author  Response | As suggested by the referee, we have updated Supplementary Table S4 to report the overlap between methods (Excerpt 2.4.3.2). |
| Excerpt 2.4.3.2 (suppl. table S4) |  |

### REF2.4.3.3 – Comparison to other methods E

| Referee  Comment | c. While we accept the rationale for using the default parameters for basicSTARRseq, using the default parameters for MACS2 is harder to justify as it was not designed for STARR-seq. This is an important point since MACS2 documentation explicitly provides different sets of optimal parameters for different types of data, since peak characteristics are different. |
| --- | --- |
| Author  Response | We thank the referee for pointing this out. We believe there is a bit of misunderstanding here. We originally used the term “default parameters” to denote a set of parameters optimized for running STARR-seq data. However, given this criticism, we refrained from using the term “default parameters” in the revised manuscript, and we updated the Methods section to clarify this (Excerpt 2.4.3).  To the best of our knowledge, there is no MACS2 documentation on choosing optimal parameters for STARR-seq datasets. However, we referenced multiple previous studies when choosing a set of optimal parameters for MACS2.   - Johnson et al., 2018 on GM12878 whole-genome STARR-seq, “…Regions were called individually for each sample using merged STARR-seq input alignments as controls with the MACS2 package using the following parameters: -f BAMPE -g hs –ratio –keep-dup all -q 0.10. The custom scaling ratio (–ratio) provided to MACS was generated for each sample using the NCIS algorithm …” <https://www.nature.com/articles/s41467-018-07607-x#Sec8> - Liu et al., 2017 on LNCaP capture STARR-seq, “… we combined data from two biological replicates and called enhancer peaks by MACS2 (default setting, q-value < 0.05) …” <https://genomebiology.biomedcentral.com/articles/10.1186/s13059-017-1345-5#Sec9> |
| Excerpt 2.4.3.3 (main text) | “…  **MACS2**  We used MACS2 version 2.1.1 [23] with the optimal parameters suggested for a human STARR-seq dataset (-f BAMPE -g hs). We also used an option to allow duplicates in read (--keep-dup all), since our STARR-seq dataset was multiplexed. We called peaks with an FDR cutoff of 0.05 (-q 0.05), as recommended by the author of the software.  …” |

### REF2.5.1 – External datasets A

| Referee  Comment | 1. The definition of “percent overlap” is confusing. Is this the percent of peaks called that exist in the validation set, or the percent of the peaks in the validation set that were called? |
| --- | --- |
| Author  Response | We revised the manuscript to clarify this (Excerpt 2.5.1). When we compared our WG-STARR-seq method to alternative enhancer assays, we used the percent of the peaks in the validation set that were called. We did this because most of the alternative enhancer assays are not genome wide. |
| Excerpt 2.5.1 (main text) | “…  Since most of the previous enhancer assays were not at a genome-wide scale and were limited in scope to specific loci, we focused on how many previously characterized enhancers were recovered using different methods.  …” |

### REF2.5.2 – External datasets B

| Referee  Comment | 2. It would be useful for the authors to report both numbers, and even construct actual ROC/PR curves using the validation set as the ground truth. This would also make the non-standard p-value threshold (0.001) and subsampling of results unnecessary. |
| --- | --- |
| Author  Response | We think there is a bit of misunderstanding. While it is always good idea to provide as much information as possible, as described earlier, we feel that it could be misleading to report a fraction of the whole-genome assay if the other enhancer assays only focused on a limited number of loci. |

### REF2.5.3 – External datasets C

| Referee  Comment | 3. Please provide a more complete description of the. Results in Figure S8: STARRPeaker correctly calls 6 of the 9 peaks, but also calls several other regions that are not peaks. The false-positive rate seems to be much higher in the competing methods, but the authors don’t mention this either. |
| --- | --- |
| Author  Response | We thank the referee for pointing this out. This section highlights the strength of our method and using the CRISPRi dataset to illustrate this point is a good suggestion. We have re-drawn Supplementary Figure S11 including more tracks (Excerpt 2.5.3.2). As the referee pointed out, these definitively show the higher false positive rate of the other methods. As suggested, we also expanded the text on this section. Furthermore, we added a new sensitivity and specificity analysis to demonstrate the superiority of the STARRPeaker method (Excerpt 2.5.3.1). Please note that former Supplementary Figure 8 has been relabeled as S11. |
| Excerpt 2.5.3.1 (main text) | “…  Second, we examined the nine distal enhancers from the GATA1 and MYC loci characterized in depth by a CRISPRi tiling screen [43]. Using the nine enhancers as the ground truth and assuming there are approximately 1,300 potential negative elements in the span of a 1.3 Mb genomic sequence, we performed sensitivity and specificity analyses of three competing methods. We think this is a substantial dataset to benchmark the performance as it provides an orthogonal line of evidence for true in-vivo enhancers and highlights that the most genomic regions are dominated by negatives. As speculated, we found that STARRPeaker had the highest specificity. While both BasicSTARRseq and MACS2 identified a few more enhancers, their false-positive rate was much higher than that of STARRPeaker (Supplementary Figure S11, Supplementary Table S8). Furthermore, upon close examination of three enhancers that were missed by STARRPeaker, we observed the coverage was much lower than other regions identified as enhancers.  …” |
| Excerpt 2.5.3.2 (suppl. fig. S11) |  |

### REF2.5.4.1 – External datasets D

| Referee  Comment | 4. Application to external datasets  a. Figure S9B seems to suggest that the results attained from basicSTARRSeq and some post-hoc filtering are superior to the STARRPeaker results, using the same enrichment metric used throughout the manuscript. This could be due to peaks that STARRPeaker missed that would increase the enrichment, or additional peaks that STARRPeaker called that are decreasing the enrichment. Please comment on this. |
| --- | --- |
| Author  Response | We appreciate the referee pointing this out. As noted, a rigorous post-hoc filtering was applied on the published dataset (P-value 1E-5 with corrected enrichment ≥ 4), which is difficult to beat especially when having more peaks. We can argue that a similar post-hoc filtering could be applied to STARRPeaker peaks, which would further increase the enrichment. To illustrate our point, we added a new Supplementary Figure 12C comparing BasicSTARRseq and STARRPeaker using the same post-hoc filtering process (Excerpt 2.5.4.1). Please note that former Supplementary Figure 9 has been relabeled as S12. |
| Excerpt 2.5.4.1 (main text) | “…  When we compared the quality of STARRPeaker peaks (n=16,150) to the published post-hoc filtered peaks (n=9,610), we found that STARRPeaker peaks were highly enriched with chromatin accessibility signals despite having 6,540 additional peaks from the same HeLa-S3 dataset (Supplementary Figure S12B). When we applied the same post-hoc filtering approach to STARRPeaker peaks, the chromatin accessibility enrichment was further improved (Supplementary Figure S12C).  …” |
| Excerpt 2.5.4.1 (suppl. fig. S12) |  |

### REF2.5.4.2 – External datasets E

| Referee  Comment | b. The authors apply STARRPeaker to a Cap-STARR-seq dataset, and report that STARRPeaker calls less than 25% of the published peaks, but provide no further exploration regarding which set of results is superior. It is then concluded that STARRPeaker can be “reliably applied” but the meaning of this term is not explained. |
| --- | --- |
| Author  Response | We have reanalyzed the Cap-STARR-seq dataset (HiDRA) and rewritten the section (Excerpt 2.5.4.2). The HiDRA dataset consists of five replicates. Previously, we were very conservative and treated each replicate independently. However, upon close examination of the published paper, we found that the authors actually merged their replicates before calculating abundances. These changes undoubtedly increase the signal-to-noise ratio. Upon reanalysis, we found an improved result showing approximately half of STARRPeaker peaks overlapping with the published results (n=26,318). |
| Excerpt 2.1 (main text) | “…  Second, we tested if STARRPeaker can be reliably applied to captured STARR-seq datasets (Cap-STARR-seq). We applied STARRPeaker to a previously characterized GM12878 STARR-seq dataset based on an ATAC-seq-capture technique called HiDRA [44] and compared its performance with published results. The HiDRA dataset was reported to have ~65,000 regions with enhancer function. In the STARRPeaker run, we identified 52,857 regions with significant enhancer activities from the five replicates they produced. Approximately half of STARRPeaker peaks overlapped with the published results (n=26,318). While it is debatable to claim that one method is superior to the other, this result clearly demonstrates that STARRPeaker can be applied to the Cap-STARR-seq dataset.  …” |

### REF2.5.4.3 – External datasets F

| Referee  Comment | c. When applying STARPeaker to a third dataset, the authors report that the method recovers a smaller set of peaks, concluding that STARRPeaker is highly specific, and more robust and reliable. However, as stated earlier in the manuscript, high specificity is often traded for low sensitivity, and it is not clear that this isn’t the case here. In addition to the peaks STARRPeaker calls, there are 149 additional peaks that are called by both competing methods but not by STARRPeaker. The authors should justify the claim that these are false positives of the other methods and not false negatives of STARRPeaker. |
| --- | --- |
| Author  Response | We think this question is better addressed using our previous section titled “Comparison to previously characterized enhancers” where we used distal enhancers identified from a CRISPRi tiling screen as the gold standard and surveyed the tradeoff between sensitivity and specificity (Excerpt 2.5.3). As the referee speculated, STARRPeaker had a higher specificity at the expense of lower sensitivity compared to other methods. However, as the example highlighted, the loss of sensitivity was minimal and the benefit of having a more specific test outweighed the shortcoming of a lower sensitivity. |

### REF2.6.1.1 – (Minor) figure caption

| Referee  Comment | 1. Figure 1:  a. “We found that the position of the peaks shifted up approximately 200 bp when we used the alternate model (Figure 1A, Supplementary Figure 1A). Such a shift caused by the read-based coverage could lead to the omission of TF binding sites located at the boundary. “I am assuming that the label “r1” in figure 1A indicates the beginning of read 1. In that case, isn’t it expected – by definition – to see a shift in the peak centers? |
| --- | --- |
| Author  Response | We think there is a bit of misunderstanding. The label “r1” in Figure 1A indicates replicate 1 rather than read 1. We revised the figure caption from r1 to rep1 in the revised manuscript to make this clearer (Excerpt 2.6.1.1). |
| Excerpt 2.6.1.1 (main fig. S1A) |  |

### REF2.6.1.2 – (Minor) clarification

| Referee  Comment | b. “peaks calculated based on the alternate model had lower fold enrichment and were less confident“ The terms fold enrichment and the procedure for estimating confidence have not been defined up to this point of the manuscript. |
| --- | --- |
| Author  Response | As suggested by the referee, we clarified the term used in the revised manuscript (Excerpt 2.6.1.2). |
| Excerpt 2.6.1.2 (main text) | “…  We found that the position of the peaks shifted up approximately 200 bp when we used the alternate model (Figure 1A, Supplementary Figure S1A). Such a shift caused by the read-based coverage could lead to the omission of TF binding sites located at the boundary. Moreover, we observed that the read-based coverage diluted the overall STARR-seq signal; as a result, peaks calculated based on the alternate model had lower fold enrichment (ratio of STARR-seq output to input) and were less confident (based on statistical significance of the identified peaks) and broader in size (Figure 1B-D, Supplementary Figure S1B-D).  …” |

### REF2.6.1.3 – (Minor) remove the less substantiated statements

| Referee  Comment | c. Generally, we find that the comparison to a “read- ends” quantification lacks in rigor. However, we do see this as a minor point. We suggest that the authors remove the less substantiated statements (e.g., “The benefit of using the center of the fragment is highlighted in Figure 1E, where we find more concise and precise peak”) and shorten this section. |
| --- | --- |
| Author  Response | As suggested by the referee, we revised the less substantiated statements. |
| Excerpt 2.6.1.3 (main text) | “…  Overall, the fragment-based coverage offered a more accurate representation of the STARR-seq readout compared to the read-based coverage counting scheme. We illustrated the benefits of using the center of the fragment in Figure 1E, which compares the average size of peaks and fold enrichments using alternate coverage counting methods.  …” |

### REF2.6.2 – (Minor) clarification

| Referee  Comment | 2. Figure 2: which dataset is being used there? |
| --- | --- |
| Author  Response | We used the HepG2 WG-STARR-seq dataset for the plot. We made it clear in the figure description and provided a shadow figure for the K562 dataset (Excerpt 2.6.2, Supplementary Figure S2). |
| Excerpt 2.6.2 (main text) | “…  **Figure 2** Confounding factors in the STARR-seq assay. STARR-seq output and input coverages are significantly correlated with **(A)** input coverage, **(B)** GC content, **(C)** mappability, and **(D)** RNA structure folding. PCC: Pearson Correlation Coefficient. Plots were from a sampling of 5,000 random genomic bins. The HepG2 dataset was used for the plot.  …” |

### REF2.6.3 – (Minor) QQ-plots

| Referee  Comment | 3. Supplementary Figure 2: the QQ-plots in panel A are not explained or discussed. How were the theoretical quantiles computed? |
| --- | --- |
| Author  Response | As suggested by the referee, we added a new section titled “Fitting of distributions to STARR-seq dataset” to the Methods section (Excerpt 2.6.3). |
| Excerpt 2.6.3 (main text) | “…  **Fitting of distributions to STARR-seq dataset**  To build a statistical model that best describes the STARR-seq readout, we tested fitting of various univariate distributions to the output coverages of the STARR-seq dataset. To eliminate the influence of input coverage on output coverage, we subsampled bins with an input coverage value of 20 (approximately a median input coverage for both the HepG2 and K562 datasets) and used their output coverage values for the underlying observed distribution. We fit binomial, Poisson, and negative binomial distributions and estimated parameters using MLE. For binomial distributions, we assumed the number of the trial as the sum of the STARR-seq input and output coverage, and the probability of success as the sum of the STARR-seq output coverage divided by the total input and output coverage, as described previously [15]. The Poisson distribution is described by a single parameter λ, whereas the negative binomial distribution is described by both μ and the shape parameter θ. For example, we found λ=41.6, μ=41.6, and θ=8.9 for the HepG2 dataset. Based on estimated parameters, we plotted the expected distribution quantiles from 0 to 100 percentiles against the actual observed quantiles. We plotted a straight diagonal line to show how well each distribution fit with the actual observed data.  …” |

### REF2.6.4 – (Minor) figure legend

| Referee  Comment | 4. Supplementary Figure 2: please define the covariate abbreviations. |
| --- | --- |
| Author  Response | As suggested by the referee, we revised the figure legend (Excerpt 2.6.4). Please note that the former Supplementary Figure has been relabeled as S3. |
| Excerpt 2.6.4 (main text) | “…  **Supplementary Figure S3** Contribution of covariates and model selection. **(A)** Q-Q plots of residuals for various models with different sets of covariates showing the goodness of fit. **(B)** Both AIC and BIC measure relative qualities of statistical models considering the trade-off between the goodness of fit and the simplicity of the model. DNA: genomic DNA fragment coverage; GC: GC-bias; MAP: mappability, FOLD: folding free energy; AIC: Akaike information criterion; BIC: Bayesian information criterion.  …” |

### REF2.6.5 – (Minor) clarification

| Referee  Comment | 5. Figures 2 and 3 seem to use a single dataset, but it is not clear which one (HepG2 or K562) and if both replicates were used or only a single one. |
| --- | --- |
| Author  Response | We used HepG2 for both Figure 2 and 3. We revised the figure legend to make this clear. Additionally, we provide a shadow figure for the K562 dataset in Supplementary Figures S2 and S4 (Excerpt 2.6.2). |

### REF2.6.6 – (Minor) clarification

| Referee  Comment | 6. Line387: “shifted toward the enrichment of sequencing reads ”. Not clear what this mean. |
| --- | --- |
| Author  Response | This is related to the shortcomings of BasicSTARRseq peaks. We clarified this statement in the revised manuscript (Excerpt 2.6.6). |
| Excerpt 2.6.6 (main text) | “…  However, we noticed that BasicSTARRseq peaks were fragmented and off-centered from the controls due to its limitations of fixed peak size and read-based coverage calculation. As a result, several false-positive peaks were called adjacent to the controls.  …” |

### REF2.6.7 – (Minor) figure axis labels

| Referee  Comment | 7. The authors replaced most instances of “STARR-seq coverage” with “output library coverage”, but this term remains in the figure labels of figures 2-4. |
| --- | --- |
| Author  Response | As suggested by the referee, we corrected the axes labels in Figures 2-4. |

### REF2.6.8 – (Minor) histone modification control

| Referee  Comment | 8. Figure 5: what is the control in the histone modification enrichment? |
| --- | --- |
| Author  Response | Each histone ChIP-seq experiment is coupled with a matching input DNA control. More specific information about the controls for each ChIP-seq experiment can be found at the ENCODE portal with accession information listed in Supplementary Table S3. |

### REF2.6.9 – (Minor) denominator

| Referee  Comment | 9. Figure 6: which numerator was used for the % overlap (20k or the number of peaks in the published/ reference dataset)? |
| --- | --- |
| Author  Response | We used the total number of peaks in each dataset as the denominator. We clarified this in the revised manuscript (Excerpt 2.6.9). |
| Excerpt 2.6.9 (main text) | “…  **Figure 6** Comparison of peaks using an external dataset for **(A)** HepG2 or **(B)** K562 cell lines. Peaks identified from STARRPeaker as well as BasicSTARRseq and MACS2 were compared against a published dataset. For a fair comparison, all peaks were centered at the summit, uniformly thresholded using P-value < 0.001, and 20,000 peaks were randomly drawn from peaks identified by each peak caller using the recommended settings. The fraction of overlap was computed for each replicate. We used the total number of peaks in each dataset as the denominator. We considered it an overlap when at least 50% of peaks intersected each other.  …” |

### REF2.6.10 – (Minor) trade-off between MPRA and STARR-seq

| Referee  Comment | 10. When discussing the comparative advantages of STARR-seq to MPRA, please mention the trade-off between the methods: STARR-seq can be made genome-wide and unbiased, but is limited to sequences that already exist in the genome, whereas MPRA is more limited in scale but enable testing of perturbed/mutated sequences, or multiple alleles in the same system. |
| --- | --- |
| Author  Response | STARR-seq has been applied to test naturally occurring mutated sequences in the past, but generally speaking, the trade-off statement holds true. We added this concept to the revised manuscript (Excerpt 2.6.10). |
| Excerpt 2.6.10 (main text) | “…  Generally, STARR-seq can be genome wide and unbiased, but the technique is limited to sequences that already exist in the genome. Although MPRA is more limited in scale, it enables testing of multiple perturbed synthetic sequences in the same system.  …” |

### REF2.6.11 – (Minor) p-value

| Referee  Comment | 11. It is stated that “the adjusted p-value should be regarded as the likelihood of a candidate region being an enhancer” (L247). This interpretation of p-values is misleading. P-values are likelihood under the null hypothesis, not under the alternative, so they would be inversely related to the likelihood of a candidate region being an enhancer. |
| --- | --- |
| Author  Response | We understand that this statement could be misleading and contradict the definition of a p-value. We rephrased the statement in the revised manuscript (Excerpt 2.6.11). |
| Excerpt 2.6.11 (main text) | “…  Note that the adjusted P-value only refers to the unlikelihood of a candidate region being an enhancer by chance while the fold enrichment can be directly interpreted as a quantitative measure of enhancer activity.  …” |

### REF2.6.12 – (Minor) typo

| Referee  Comment | 12. (L307) The term \hat{beta} seems to be a typo. |
| --- | --- |
| Author  Response | We thank the referee for finding this error. We corrected it in the revised manuscript. |

### REF2.6.13 – (Minor) correlation

| Referee  Comment | 13. In Supplemental Figure 3, the authors show a high correlation between replicates, but it is not clear what exactly is correlated. Is this correlation of effect size after peak-calling using STARRPeaker, or correlation of the data itself? How was this computed? |
| --- | --- |
| Author  Response | Correlation is calculated based on RNA fragment counts across genomic bins. We made this clear in the revised manuscript (Excerpt 2.6.13). Please note that former Supplementary Figure 3 has been relabeled as S6. |
| Excerpt 2.6.13 (main text) | “…  We processed two biological replicates from each cell type independently and assessed the correlation between each pair (see Methods for detail). We identified 50,389 and 52,927 candidate enhancers from HepG2 replicate 1 and 2, and by consolidating peaks from both replicates, we identified 32,929 (65.3% of rep 1; 62.2% of rep2) reproducible candidate enhancers from the HepG2 cell line. Similarly, by consolidating 30,194 and 41,810 candidate enhancers from K562 replicate 1 and 2, we identified 20,471 (67.8% of rep 1; 49.0% of rep 2) reproducible candidate enhancers from the K562 cell line (Supplementary Table S1). Overall, we observed high correlation of RNA fragment coverage between two replicates (PCC=0.99 for both HepG2 and K562; see Supplementary Figure S6). This result indicates the correlation is dominated by negatives.  …  **Supplementary Figure S6** Correlation of RNA fragment coverage between replicates for **(A)** HepG2 or **(B)** K562 cell lines. Pairwise coverage was calculated using 1,000 bp bins across the genome. The X- and Y-axis represent the natural log of fragment counts. A pseudo count of 1 was added to the fragment counts for plotting only. Bins with abnormally high fragment counts were removed to avoid inflation of the Pearson correlation. We used the median absolute deviation method with a scaling factor of 200 to filter extremely large deviations from the median.  …” |

### REF2.6.14 – (Minor) alternate mode

| Referee  Comment | 14. An alternative mode is discussed in L285. While this explanation was revised, it is still unclear what the benefit of this alternative mode, when should users consider using it, and how it compares with the default mode. The authors should either expand on this point or avoid mentioning this alternative entirely. |
| --- | --- |
| Author  Response | As suggested by the referee, we added several example scenarios where the alternate mode could offer benefits to the revised manuscript (Excerpt 2.6.14). In summary, the alternate mode is useful for STARR-seq libraries that only contain discrete genomic DNA elements and users who want to ignore the effect of covariates. |
| Excerpt 2.6.14 (main text) | “…  Alternatively, instead of using the input library variable $t_{i}$ as one covariate, we can directly use it as an offset variable. Generally, a fractional observation cannot be modeled using discrete probability. However, an offset variable in a generalized linear model can be used to correct the response term to behave like a fraction. One advantage of using the input variable as an “exposure” to the RNA output coverage is that it allows us to directly model the basal transcription rate (the ratio of RNA to DNA) as a rate response variable. This mode could be beneficial in multiple scenarios. First, for a STARR-seq dataset in which the guide DNA library only contains discrete elements, direct modeling of the basal transcription rate would provide a more accurate measure of activity, especially in the absence of readouts from adjacent regions. Second, if a user ignores the effect of covariate, this mode simplifies the model and provides peaks purely based on the ratio of RNA to DNA. More details on this alternative parametrization are included in the Methods section. In our STARRPeaker model, we used four covariates; fragment coverage of input genomic libraries, GC content, mappability, and the thermodynamic stability of genomic libraries.  …” |

References

1. Pennacchio LA, Bickmore W, Dean A, Nobrega MA, Bejerano G. Enhancers: five essential questions. Nat Rev Genet [Internet]. NIH Public Access; 2013 [cited 2019 Sep 28];14:288–95. Available from: http://www.ncbi.nlm.nih.gov/pubmed/23503198

2. Muerdter F, Boryń ŁM, Woodfin AR, Neumayr C, Rath M, Zabidi MA, et al. Resolving systematic errors in widely used enhancer activity assays in human cells. Nat Methods [Internet]. Nature Publishing Group; 2018 [cited 2019 Sep 11];15:141–9. Available from: http://www.nature.com/articles/nmeth.4534

3. Liu Y, Yu S, Dhiman VK, Brunetti T, Eckart H, White KP. Functional assessment of human enhancer activities using whole-genome STARR-sequencing. Genome Biol [Internet]. 2017 [cited 2019 Jul 5];18:219. Available from: https://genomebiology.biomedcentral.com/articles/10.1186/s13059-017-1345-5

4. Wang X, He L, Goggin SM, Saadat A, Wang L, Sinnott-Armstrong N, et al. High-resolution genome-wide functional dissection of transcriptional regulatory regions and nucleotides in human. Nat Commun. Nature Publishing Group; 2018;9:1–15.

5. Benjamini Y, Hochberg Y. Controlling the false discovery rate: a practical and powerful approach to multiple testing. J R Stat Soc Ser B [Internet]. John Wiley & Sons, Ltd (10.1111); 1995 [cited 2019 Jul 5];57:289–300. Available from: http://doi.wiley.com/10.1111/j.2517-6161.1995.tb02031.x

6. Huang L, Zhang H, Deng D, Zhao K, Liu K, Hendrix DA, et al. LinearFold: linear-time approximate RNA folding by 5’-to-3’ dynamic programming and beam search. Bioinformatics [Internet]. Narnia; 2019 [cited 2019 Sep 11];35:i295–304. Available from: https://academic.oup.com/bioinformatics/article/35/14/i295/5529205

7. Lorenz R, Bernhart SH, Höner zu Siederdissen C, Tafer H, Flamm C, Stadler PF, et al. ViennaRNA Package 2.0. Algorithms Mol Biol [Internet]. BioMed Central; 2011 [cited 2020 Mar 13];6:26. Available from: https://almob.biomedcentral.com/articles/10.1186/1748-7188-6-26

8. Mathews DH, Disney MD, Childs JL, Schroeder SJ, Zuker M, Turner DH. Incorporating chemical modification constraints into a dynamic programming algorithm for prediction of RNA secondary structure. Proc Natl Acad Sci U S A. National Academy of Sciences; 2004;101:7287–92.

9. Papoulis A, Athanasios. Probability, random variables and stochastic processes. New York McGraw-Hill, 1984, 2nd ed [Internet]. 1984 [cited 2019 Jul 5]; Available from: http://adsabs.harvard.edu/abs/1984prvs.book.....P

10. Pang B, Snyder MP. Systematic identification of silencers in human cells. Nat Genet [Internet]. Nature Publishing Group; 2020 [cited 2020 Mar 13];52:1–10. Available from: http://www.nature.com/articles/s41588-020-0578-5

11. Kawaji H, Kasukawa T, Forrest A, Carninci P. The FANTOM 5 collection, a data series underpinning mammalian transcriptome atlases in diverse cell types. Sci Data. 2017;2016–8.

12. Inoue F, Kircher M, Martin B, Cooper GM, Witten DM, McManus MT, et al. A systematic comparison reveals substantial differences in chromosomal versus episomal encoding of enhancer activity. Genome Res [Internet]. 2017 [cited 2019 Jul 6];27:38–52. Available from: http://www.ncbi.nlm.nih.gov/pubmed/27831498

13. Klein JC, Agarwal V, Inoue F, Keith A, Martin B, Kircher M, et al. A systematic evaluation of the design, orientation, and sequence context dependencies of massively parallel reporter assays. bioRxiv [Internet]. Cold Spring Harbor Laboratory; 2019 [cited 2019 Mar 15];576405. Available from: https://www.biorxiv.org/content/10.1101/576405v1

14. Rathert P, Roth M, Neumann T, Muerdter F, Roe J-S, Muhar M, et al. Transcriptional plasticity promotes primary and acquired resistance to BET inhibition. Nature [Internet]. 2015 [cited 2019 Jul 5];525:543–7. Available from: http://www.ncbi.nlm.nih.gov/pubmed/26367798

15. Cameron ACA, Trivedi PK. Regression Analysis of Count Data [Internet]. Cambridge: Cambridge University Press; 2013 [cited 2019 Jul 5]. Available from: http://ebooks.cambridge.org/ref/id/CBO9781139013567
